# Versatile Organic Electrochemical Transistors with Self-Assembled Coronene Nanofiber Arrays for the Isolation and Detection of Circulating Tumor Cells and Enhanced Secretion of Extracellular Vesicles

**DOI:** 10.1021/acsami.5c05442

**Published:** 2025-05-30

**Authors:** Edgar Daniel Quiñones, Jiashing Yu, Rou-Zhen Liu, Yi-Shiuan Li, Yu-Chuan Lu, Yu-Sheng Hsiao

**Affiliations:** † Department of Materials Science and Engineering, 34878 National Taiwan University of Science and Technology, Taipei 106335, Taiwan; ‡ Taiwan International Graduate Program, Sustainable Chemical Science & Technology, Academia Sinica, Institute of Chemistry, Taipei, 115, Taiwan; § Department of Chemical Engineering, 33561 National Taiwan University, Taipei 10617, Taiwan; ∥ Department of Materials Engineering, 56082 Ming Chi University of Technology, New Taipei City 24301, Taiwan; ⊥ Department of Surgical Oncology, National Taiwan University Cancer Center, 587787 National Taiwan University College of Medicine, Taipei 10672, Taiwan; # Department of Urology, National Taiwan University Hospital, Taipei 100225, Taiwan; ¶ Energy and Sustainability Tech Graduate Institute, National Taiwan University of Science and Technology, Taipei 106335, Taiwan

**Keywords:** bioelectronic interfaces (BEIs), organic electrochemical transistor (OECT), nanofiber arrays (NFAs), poly(3,4-ethylenedioxythiophene):polystyrenesulfonate (PEDOT:PSS), circulating tumor cells (CTCs), extracellular vesicle (EV)

## Abstract

The integration of organic bioelectronic interfaces has opened new avenues for merging biological systems with electronics, offering transformative solutions to challenges in both fields. In this study, we present a novel hybrid organic electrochemical transistor (OECT) featuring self-assembled three-dimensional (3D) nanofiber arrays (NFAs) of small-molecule semiconductors grown on poly­(3,4-ethylenedioxythiophene):polystyrenesulfonate active-layer channels. The hybrid OECT was further evaluated for its potential to capture, recover, and biosense circulating tumor cells (CTCs), as well as to enhance extracellular vesicle (EV) secretion through electrical stimulation (ES). Using template-free self-assembly via standard thermal evaporation, out-of-plane coronene (CR)-based NFAs were fabricated and surface-engineered with 1-pyrenebutyric acid through π–π interactions, enabling bioaffinity coatings on OECTs for advanced biological applications. The device demonstrated efficient CTC isolation, achieving an isolation rate exceeding 90% for MCF-7 breast cancer cells spiked into THP-1 monocytic cells (10^6^ cells mL^–1^), with minimal nonspecific binding by incorporating a specific antibody-coated CR-based NFA layer. Moreover, over 80% of the captured CTCs were released from CR-based NFAs during cyclic voltammetry sweeps in phosphate-buffered saline. The device also enhanced EV secretion by incorporating a collagen-coated CR-based NFA layer, which supported cell attachment and proliferation under sustained ES at 20 V, 0.5 Hz, with 5 ms pulses for 24–72 h periods. EV production increased by approximately 12.4-fold in MCF-7 cells and 8.0-fold in immortalized bone marrow stromal cells without significantly altering the EV size or requiring additional cellular modifications. This dual-functionality platform, enabled by a surface-engineered 3D-hybrid OECT, is a powerful tool for selective CTC isolation, liquid biopsy purification, and enhanced EV production. This versatility highlights the potential of this approach for advanced bioelectronic applications and paves the way for innovations in diagnostics and therapeutic research.

## Graphical abstract

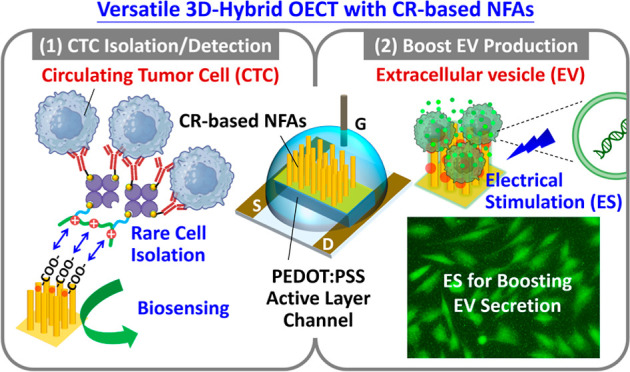

## 1 Introduction

Organic bioelectronic interfaces (OBEIs) have demonstrated utility in wide-ranging biomedical applications and play a pivotal role in many scientific fields. However, their limitations have become increasingly evident, particularly as the demand for relatively small and more controllable designs and geometries continues to increase. Moreover, the increasing use of thin films composed of π-stacked small molecule semiconductors in surface engineering techniques based on topographical and chemical approaches has gained considerable attention. This trend is particularly pronounced in the development of OBEI devices, presenting unique requirements and challenges.^1–4^ In advanced biomedical device applications, research has emphasized the integration of three-dimensional (3D)-OBEIs with electrodes or the active layers of electronic components or both. These 3D-OBEIs, characterized by their mixed electronic and ionic conductive properties, act as innovative intermediate layers that enable the bidirectional coupling of electrons and ions. Moreover, they enhance structural integrity and functionality, facilitating seamless communication between biological and electronic systems.^5,6^ Consequently, OBEIs offer several notable advantages, including ease of chemical modification, low impedance between the electrode and the aqueous electrolyte, and compatibility with low-temperature processing. They are suited for mass production and provide remarkable versatility in surface adjustments, enabling the dynamic control of electrochemical, optical, and mechanical properties through doping and dedoping processes.^7,8^

An efficient approach to producing small organic molecule semiconductor (SOMS)-based nanostructures involves precise control of deposition conditions, including fine-tuning surface energies and substrate temperatures during the standard thermal evaporation processes.^9–12^ This method has enabled extensive research into the self-assembly of SOMSs, facilitating the creation of various nanostructures for organic electronic applications. For example, organic nanocrystals forming SOMSs demonstrate enhanced electrical and optical performance because of their low defect densities, attributable to self-assembly or crystallinity or both, as well as their quantum size effects and ease of doping at the material level.^13–15^ Despite their promise, the practical applications of SOMSs in cell-based bioelectronics remain limited. Furthermore, π-stacked SOMSs often fall short in several aspects, such as biocompatibility, flexibility, and biostability. To address these shortcomings, they are typically paired with complementary materials.^16,17^ Among the various materials used in OBEIs, electrically conducting polymers (CPs), such as polypyrrole and poly­(3,4-ethylenedioxythiophene) (PEDOT), have emerged as leading candidates. Their prominence stems from their intrinsic electrical properties, including efficient charge transport, charge–discharge behavior, responsiveness, high biocompatibility, and mechanical flexibility. In addition, research has demonstrated that topographical and biochemical modifications of these materials can notably impact key cellular activities, such as differentiation, proliferation, and adhesion.^18–21^ Furthermore, CPs hold great potential as the active-layer channels of organic electrochemical transistors (OECTs) for biosensing applications, broadening their utility in bioelectronics. Central to these advancements is the ability to manipulate the 3D structures of OBEIs, thereby enhancing their efficacy in cell and tissue regulation and expanding their roles in diagnostic and therapeutic applications, particularly in noninvasive liquid biopsy applications. These findings suggest that integrating SOMSs with CPs can yield synergistic benefits, expanding their utility across various bioelectronic applications.^6,22^

Isolating circulating tumor cells (CTCs) is invaluable for diagnosing cancer and monitoring disease progression. These cells detach and are released from solid primary tumors, enter the peripheral bloodstream, and potentially initiate distant metastasis. Analyzing the phenotypes and molecular characteristics of CTCs in blood-based liquid biopsies offers critical insights into tumor-specific changes over time and aids in assessing treatment efficacy. However, despite the adaptability of liquid biopsy techniques for various cancer diagnostics, identifying and isolating CTCs from blood samples remains a considerable challenge because of their exceptionally low abundance, with only a few hundred CTCs per billion blood cells.^23–27^

Recent research on nanostructures has shown that combining surface modification with bioaffinity approaches can considerably enhance the recognition and capture efficiency of CTCs with high specificity.^28–36^ This combination expands the repertoire of strategies beyond conventional methods that rely solely on individual physical properties, such as size, density, electric charge, and deformability, or biological properties, such as surface conjugation with specific biomolecules. Examples of such nanostructured materials include vertically oriented (out-of-plane) silicon nanopillars,^28,29^ nanostructured arrays of polymers,^30,31^ and horizontally oriented (in-plane) nanostructures prepared from materials, such as poly­(lactic-*co*-glycolic acid).^32^ Combined with NanoVelcro Chip assays, these configurations enable enhanced cell–substrate interactions, leading to highly efficient cancer cell isolation from liquid biopsy.^33^ Furthermore, integrating 3D-OBEIs, including CPs, nanocarbon-based materials, and SOMSs, into such chips promises marked advancements in the application of bioelectronics for cancer-related research.^34–36^

Exosomes, a type of extracellular vesicle (EV), are lipid bilayer structures of 30–150 nm in diameter secreted by various cell types. They play both beneficial and detrimental roles in human health through their participation in intercellular communication, cell-to-cell signaling, and various intracellular processes primarily achieved through the delivery of proteins and genetic information between cells. Owing to their ability to transport endogenous cargo for cell-to-cell communication, exosomes have been widely used as bioshuttles for efficiently delivering biomolecules within cells. They have also emerged as effective delivery vehicles for promoting cell proliferation in therapeutic contexts and have a notable role in advancing gene therapies.^37–40^ Exosomes and their modifications are anticipated to become valuable tools for disease treatment. The elucidation of the mechanisms underlying their production and secretion has been identified as a critical target for developing new therapies and improving their therapeutic potential.^41,42^ Laboratories typically use conditioned medium from cultured cells, followed by stepwise ultracentrifugation to isolate exosomes for research. However, this method often results in low yield and limited exosome purity. The functions, properties, and production of EVs, including exosomes, vary considerably among donor cell types. For example, mesenchymal stem cells (MSCs) have been reported to secrete EVs at rates 100–1000 times higher than those in other cell lines, such as myoblasts. Additionally, variations in protein content and EV particle number have been observed across different cell lines, which can complicate efforts to elucidate specific EV functions and optimize their applications. Therefore, increasing the yield of EVs from diverse cell sources is critical for advancing EV research. Various methods, such as affinity-based purification and polymeric precipitation, have been explored to improve the yield and purity of EVs. Moreover, developing strategies to enhance EV secretion from cells offers an avenue for boosting EV production yield.^43–46^

In this study, we developed a novel hybrid OECT featuring coronene (CR) nanofiber arrays (NFAs) grown on a PEDOT:polystyrenesulfonate (PSS) active-layer channel. This design integrates two parallel OBEI functions: isolation, recovery, and detection of CTCs and enhanced EV production during device operations under electrical stimulation (ES). The CR-based NFAs were fabricated using a template-free self-assembly approach via standard thermal evaporation, which enabled straightforward surface engineering. These NFAs were subsequently coated with 1-pyrenebutyric acid (PBA) through π–π interactions, creating bioaffinity coatings suitable for advanced biological applications. The hybrid 3D-OECT device was designed to leverage the synergistic effects of topographical and biochemical approaches. This resulted in the highly efficient capture of targeted MCF-7 cancer cells from human monocytic cell line (THP-1) cell solutions (10^6^ cells mL^–1^). In addition, the device demonstrated the ability to enhance EV secretion from cancer or stem cells under ES conditions. The hybrid 3D-OECT demonstrated enhanced biosensing performance, attributed to its improved mixed electronic-ionic conductivity and increased charge capacity density (CCD), allowing for the precise quantification of captured cancer cell densities. This versatile hybrid 3D-OECT assay exhibits considerable potential for advanced bioelectronic applications and opens new avenues for innovation in diagnostic and therapeutic research.

## 2 Experimental Section

### 2.1 Fabrication of CR-Based NFAs and OECT Devices

The indium–tin oxide (ITO) glass substrates (7 Ω sq^–1^; Ruilong Optical, Miaoli, Taiwan) were initially cut into two varying sizes to suit different applications. For OECTs designed for CTC capture and release, the substrates used were 3 cm × 3 cm in size, whereas for OECTs used for EV enhancement, the substrates used were 9.5 cm × 9.5 cm in size. The patterned ITO electrodes were fabricated following a systematic process that included wet-bench cleaning, air-plasma treatment, tape masking, laser engraving, and wet etching using a 37% HCl solution under established protocols. To ensure thorough cleaning, the wet-bench cleaning of substrates involved sequential ultrasonic agitation in acetone, ethanol, and deionized (DI) water, followed by an air-plasma treatment for 10 min (PDC-32G-2; Harrick Plasma, Ithaca, NY, USA) at a radio frequency power of 18 W to remove residual contaminants. High-density polyester electrical tape (1350F-1; 3 M Co. Ltd., Minnesota, USA) was used to mask specific patterns on the ITO substrate. The desired patterned areas were created using a commercial CO_2_ laser engraving system (Universal VLS2.30; Universal Laser System, Scottsdale, AZ, USA), and the substrates underwent wet etching with 37% HCl to finalize the electrode patterning (Figure 1a,f). Subsequently, the patterned ITO substrates were subjected to an air-plasma treatment (10 min, 18 W) to enhance their hydrophilic properties and adhesion for the subsequent spin-coating of the PEDOT:PSS materials. An aqueous solution of PEDOT:PSS was prepared by mixing 94 wt % of PEDOT:PSS (PH1000; Heraeus, Germany), 5.0 wt % dimethyl sulfoxide (DMSO; Sigma-Aldrich), and 1.0 wt % (3-glycidyloxypropyl)­trimethoxysilane (GOPS; Sigma-Aldrich). This solution was spin-coated onto the patterned ITO substrates at 2000 rpm for 30 s. The coated substrates were then annealed at 130 °C for an hour to achieve thermal cross-linking and surface dehydration (Figure 1b,g). Finally, the patterned PEDOT:PSS active-layer channels for CTC- and EV-specific applications were defined using a CO_2_ laser engraving system to remove unwanted areas (Figure 1c,h, respectively). The channels of our OECT devices were designed with specific geometries tailored to their respective functions and assessments. For transfer characteristics (*I*_d_ *–V*_g_) measurements and corresponding transconductance (*g*_m_) peak analysis, the device featured a channel length of 0.3 mm, a width of 14 mm, and a PEDOT:PSS film thickness of approximately 200 nm. For long-term (*I*_d_ *–*time) stability assessments and biosensing of the cell density differences, the channel was configured with a length of 5 mm, a width of 1.5 mm, and the same PEDOT:PSS thickness of approximately 200 nm (Figure S1a). For EV enhancement, the OECT device employed interdigital ITO electrodes (20 pairs) with a channel length of 0.8 mm, a total width of 78 mm, and a PEDOT:PSS film thickness of approximately 500 nm. Additional details regarding the EV enhancement device dimensions, as well as the corresponding optical images, are provided in Figure S1b,c.

**Figure 1:**
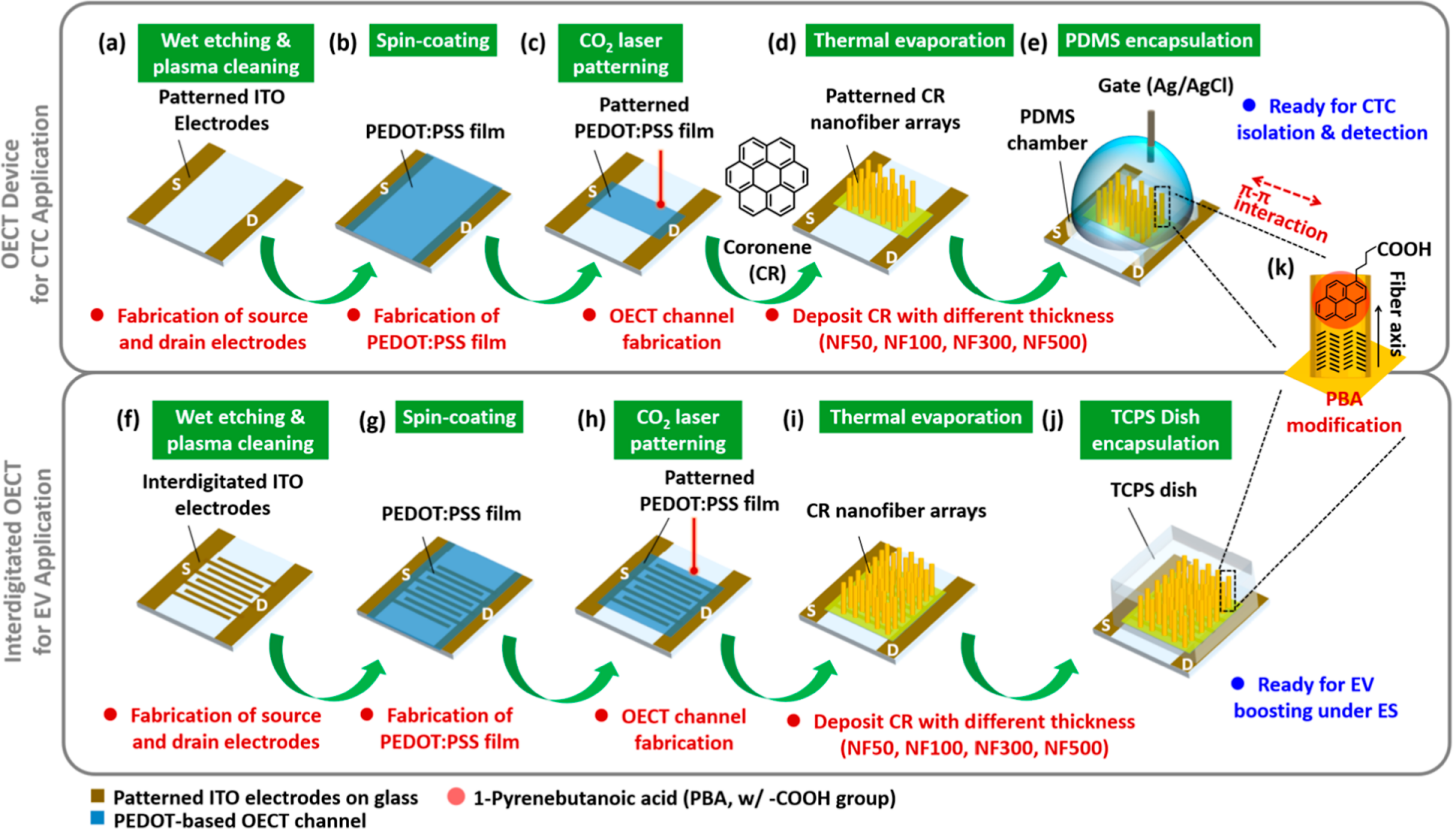
Schematic illustration of the fabrication process for a hybrid 3D-OECT device featuring a CR-based NFA coating architecture and subsequent PBA modification for (a–e) CTC- and (f–j) EV-specific applications. (a,f) Wet etching and plasma cleaning of patterned ITO substrates. (b,g) Spin-coating of PEDOT:PSS onto the substrates. (c,h) CO_2_ laser patterning of the PEDOT:PSS film for defining the active-layer channel for OECT devices. (d,i) Self-assembly and deposition of CR-based NFAs onto the PEDOT:PSS active-layer channel via thermal evaporation, forming the hybrid 3D-OECT devices. (e,j) Encapsulation using PDMS and TCPS chambers for CTC isolation/detection and EV boosting production applications, respectively. (k) PBA-based modification of 3D-OECT device through the π–π stacking interaction. **3D-OECT**: three-dimensional organic electrochemical transistor; **CR**: coronene; **NFA**, nanofiber array; **PBA**: 1-pyrenebutyric acid; **CTC**: circulating tumor cell; **EV**: extracellular vesicle; **ITO**: indium–tin oxide; **PEDOT:PSS**: poly­(3,4-ethylenedioxythiophene):polystyrenesulfonate; **PDMS**: polydimethylsiloxane; **TCPS**: tissue culture polystyrene.

After fabricating the PEDOT:PSS-based OECT devices, CR (purity: 97%; Sigma-Aldrich)-based NFAs were directly deposited onto the PEDOT:PSS active-layer channels using a shadow mask for thermal evaporation without additional purification. The process was performed under a base pressure of 3 × 10^–6^ Torr, with the substrate temperature controlled. The crucible temperature for sublimating CR materials was maintained at 120 °C. For all samples, the deposition rate was set to 1 A° s^–1^, which was controlled and monitored using a quartz crystal microbalance (QCM). The CR layer thicknesses, which were also observed via QCM, were approximately 50, 100, 300, and 500 nm. Following the deposition of CR-based NFAs onto the PEDOT:PSS films, the devices were encapsulated either within a custom-made polydimethylsiloxane (PDMS) chamber for CTC-specific applications or in a tissue culture polystyrene dish for EV-specific applications, as shown in Figure 1d–e,i–j, respectively.

### 2.2 Morphological and Electrochemical Characterizations

The morphological changes, crystalline structures, water contact angles (WCAs), and chemical configurations of CR-based NFAs were characterized using field-emission scanning electron microscopy (SEM), grazing-incidence X-ray diffraction (GIXRD), geometric mean approximation, and X-ray photoelectron spectroscopy (XPS), respectively, with their detailed results provided in the Supporting Information. Moreover, the results of the electrochemical characterization analyses, including cyclic voltammetry (CV) and electrochemical impedance spectroscopy (EIS), of the samples are also provided in the Supporting Information.

### 2.3 Surface Modification of 3D-OECT Devices

The details of the surface modification process of 3D-OECT devices and CTC isolation and release experiments are described in the Supporting Information. For EV production experiments, the 3D-OECT devices underwent an additional overnight ultraviolet (UV) cleansing process to eliminate potential sources of contamination during cell culture. After the UV treatment, the devices were carefully washed once with sterilized DI water. Subsequently, the devices were incubated with a collagen modification solution [0.1 mg mL^–1^ in 1× phosphate-buffered saline (PBS)] for an hour and then thoroughly rinsed with sterilized DI water to remove any excess nonadsorbed collagen. Finally, the devices were incubated in alpha-minimal essential medium (α-MEM) with 1% penicillin/streptomycin/amphotericin B (Thermo Fisher Scientific, Waltham, MA, USA) for at least an hour for stabilization. After the medium was removed, the EV production devices were kept in PBS and ready for use in cell culture experiments.

## 3 Results and Discussion

Utilizing data on self-assembled CR-based NFAs on large-scale reduced graphene oxide coatings produced by conventional thermal evaporation processes, 3D-OBEIs can be optimized as functional electrodes to efficiently capture rare CTCs from liquid biopsies and release them under ES, facilitating downstream analyses.^27^ In the present study, we found that CR-based NFAs could also be grown on PEDOT:PSS films formulated with 1.0 wt % GOPS and 5.0 wt % DMSO, a composition well suited for forming the active-layer channel of OECTs. Therefore, we developed a strategy for fabricating hybrid 3D-OECT devices. In this device, we incorporated patterned ITO as the source and drain electrodes, a PEDOT:PSS film as an active-layer channel, and nanostructured CR-based NFA thin films as a decoration layer, enabling fundamental device operation for biosensing while achieving enhanced cell capture and release performance and improved EV production. Our proposed method for fabricating CR-based NFAs on the PEDOT: PSS films as the active-layer channel of OECTs is illustrated in Figure 1. This method can be readily utilized for further studies on rare cell capture/release (Figure 1a–e) and EV secretion by cancer or stem cells (Figure 1f–j). The fabrication process of the OECT devices can be broadly divided into three main steps: (1) ITO patterning, wherein the source and drain electrodes were created by tape masking, CO_2_ laser engraving, and wet etching with a 37% HCl solution; (2) spin-coating, wherein a PEDOT:PSS solution was spin-coated onto the patterned ITO electrode substrate; and (3) CR-based NFA deposition, wherein small CR molecules were deposited via thermal evaporation. These constitute the primary preparation steps for OECT devices. Subsequently, two distinctive coatings were applied to the CR-based NFAs: capture CTCs through NanoVelcro chip assays, release CTCs under CV sweeping, and enhance EV production under ES operation.

Various deposition conditions were explored to investigate the morphological changes in resulting CR-based NFAs. The deposited parameters of CR materials were monitored using a QCM with a deposition rate of 1 Å s^–1^, and the thicknesses of 50, 100, 300, and 500 nm were achieved via conventional thermal evaporation at a controlled substrate temperature (*T*_sub_) of 25 °C. The samples were designated as **NF50**, **NF100**, **NF300,** and **NF500**, where the number following “**NF**” corresponds to the deposition thickness of CR-based NFAs, as monitored by QCM. As shown in Figure 2a–d, the top-view SEM images of various CR-based NFAs are presented and arranged based on their nanofiber diameter (*D*) and length (*L*) distributions. The average nanofiber diameters for **NF50**, **NF100**, **NF300**, and **NF500** were 73 ± 11 nm, 80 ± 21 nm, 107 ± 19 nm, and 151 ± 51 nm, respectively (Figure 2e–h). Similarly, the average fiber lengths were 480 ± 191 nm, 789 ± 292 nm, 1114 ± 272 nm, and 12,341 ± 435 nm, respectively (Figure 2i–l). Additionally, the density of NFAs increased progressively from **NF50** to **NF100**, **NF300**, and **NF500**. Further analysis of the cross-sectional SEM images revealed that CR-based NFAs on the PEDOT:PSS films were formed on a uniform underlying layer. This assembly was associated with an initial layer-by-layer growth followed by a Stranski–Krastanov-type growth process (Figure 3a–d), consistent with previously observed growth morphologies on reduced graphene oxide-coated ITO electrodes.^34^ The thickness of the underlying layers was closely similar, measuring 119 ± 15 nm, 129 ± 14 nm, 130 ± 19 nm, and 131 ± 10 nm, for **NF50**, **NF100**, **NF300**, and **NF500**, respectively (Figure 3e).

**Figure 2:**
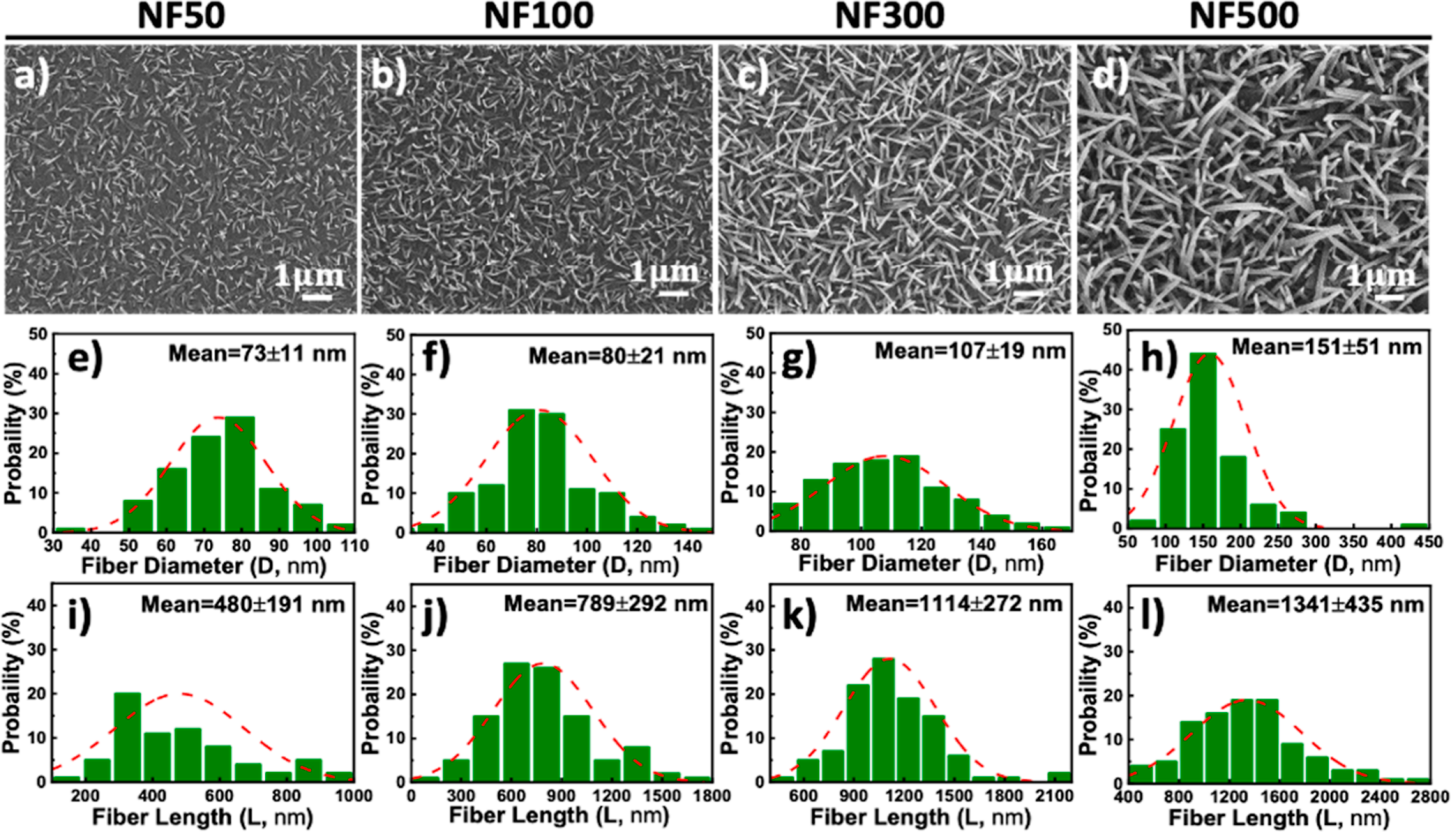
(a–d) Top-view SEM images of CR-based NFAs deposited on the PEDOT:PSS (P) films at a controlled substrate temperature of 25 °C, designated as **NF50**, **NF100**, **NF300**, and **NF500**, corresponding to deposition thickness monitored by QCM. The statistical size distributions of (e–h) fiber diameters, and (i–l) fiber lengths for the respective samples.

**Figure 3:**
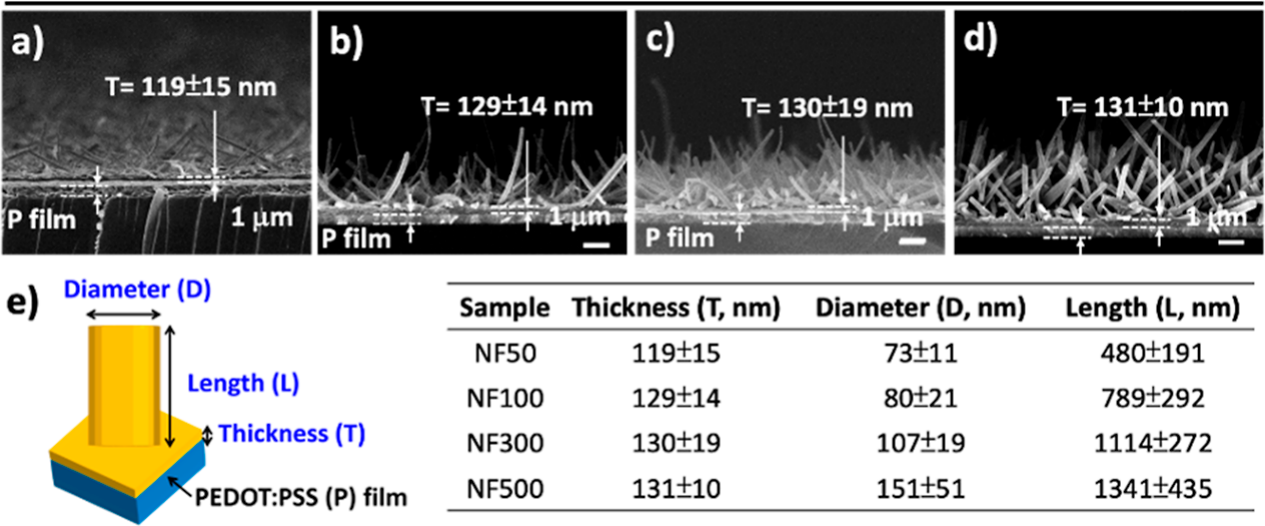
(a–d) Cross-sectional SEM images of CR-based NFA films deposited on PEDOT:PSS (P) films at a controlled substrate temperature of 25 °C, designated as **NF50**, **NF100**, **NF300**, and **NF500**, corresponding to deposition thickness monitored by QCM. (e) Statistical analysis of the thickness (*T*), diameter (*D*), and length (*L*) of CR-based NFA films.

The GIXRD spectra of the PEDOT:PSS-coated glass, CR powders, and CR-based NFA films formed on the PEDOT:PSS-coated glass substrates through thermal evaporation at *T*_sub_ = 25 °C are shown in Figure 4a–f. The broad peak observed in the spectra for the PEDOT:PSS film was indexed at 2θ of 23.8°. The proposed “alternate lamellar stacking distance of PEDOT and PSS” may stem from a structural model in which flat PEDOT (∼7.5 Å) and PSS (∼15.5 Å) layers are arranged side by side, forming an overall periodicity of approximately 23 Å. This is consistent with previous findings that moderate to high cross-linking and additive levels disrupt long-range lamellar ordering, leading to attenuation and broadening of the π–π stacking signal near 23°.^5,22,47^ The spectrum of the CR powder was indexed to the crystal structures of β- and γ-CR, indicating that it was a polycrystalline material.^48^ The peaks associated with γ-CR, corresponding to the (101̅) and (002) planes, appeared at 2θ of 9.30° and 11.90°, respectively, as indicated by blue triangles. Moreover, an emergent peak, attributed to β-CR and marked by a red rhombus, was observed at 2θ of 10.6°. This peak was consistently observed for the **NF10**, **NF50**, **NF100**, and **NF300** samples. The intensities of the peaks associated with both β- and γ-CR in NFAs on the PEDOT: PSS films increased progressively from **NF10** to **NF50**, **NF100**, and **NF300**, reflecting changes in their crystalline structure with increasing deposition thickness.

**Figure 4:**
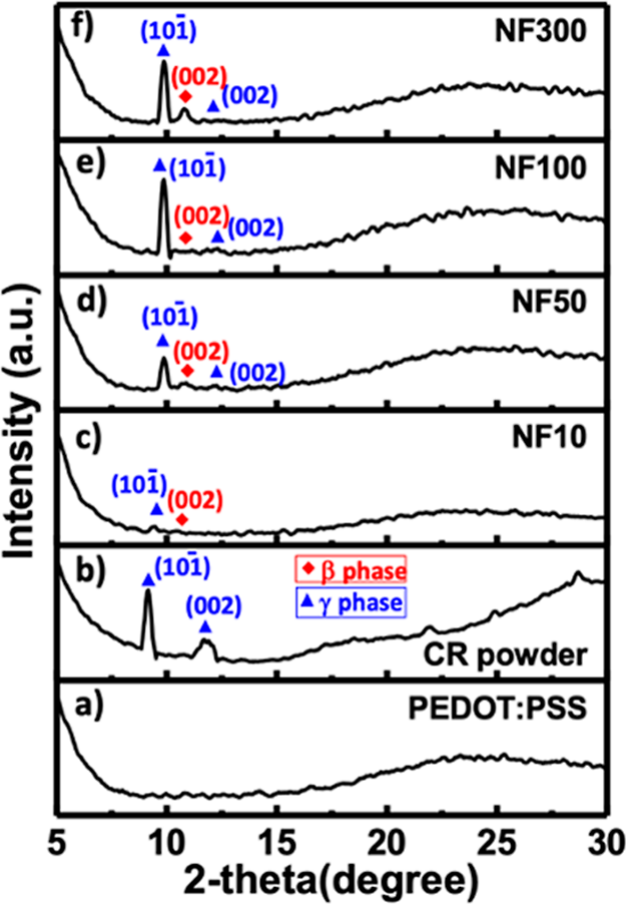
GIXRD spectra of the (a) pristine PEDOT: PSS film, (b) CR powders, and (c–f) CR-based NFA films deposited on PEDOT: PSS films at a controlled substrate temperature of 25 °C, labeled as **NF10**, **NF50**, **NF100**, and **NF300**, respectively.

To demonstrate the applicability of self-assembled CR-based NFAs in OECT device architectures, the pristine PEDOT: PSS film (**P**) and PBA-coated **NF10**, **NF50**, **NF100**, **NF300**, and **NF500** films were prepared as the active-layer channels of OECTs. The corresponding output characteristics are shown in Figure 5. The PBA coating is essential for enhancing the hydrophilicity of the CR-based NFAs thin films (Figure 1k), thereby facilitating ionic transport in and out of the underlying **P** film during electrochemical operation, as previously reported.^34^ Notably, OECT based on **NF100** exhibited the highest drain current (*I*_d_) and transconductance (*g*_m_), whereas source–drain potential (*V*_d_) remained fixed at 0.1 V (Figure 5a,b, respectively). A relatively high *g*_m_ value indicates superior amplification performance for use in biosensing and improved ES capability for cell manipulation. Although the precise mechanism underlying the enhanced *g*_m_ remains unclear, our findings confirmed that optimizing the deposition conditions for CR-based NFAs on **P** films (**NF100**) significantly improved the output performance of OECTs.

**Figure 5:**
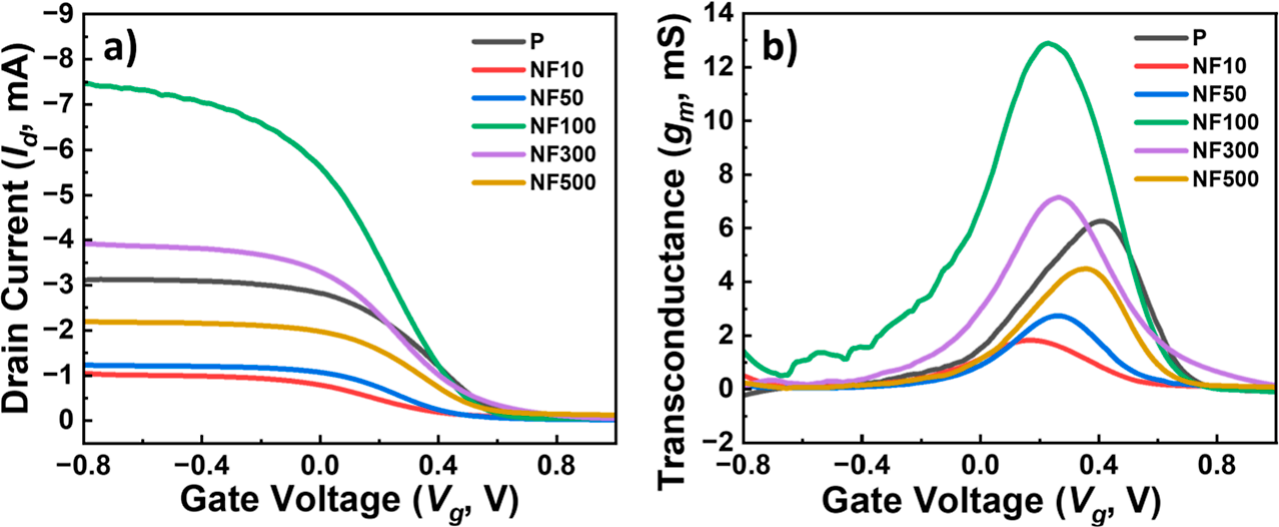
Output characteristics of OECTs with a pristine PEDOT: PSS film (**P**) and a CR-based NFA film deposited on PEDOT:PSS films (**NF10**, **NF50**, **NF100**, **NF300**, and **NF500**) as active-layer channels. (a) Transfer characteristics (*I*_d_–*V*_g_) and (b) corresponding transconductance (*g*_m_) peak curves.

Following these results, WCA analyses were performed on the pristine PEDOT:PSS film (**P**) and **NF100** to refine and verify the feasibility of PBA coating on CR-based NFAs by assessing the wettability of OECT active-layer channels. As shown in Figure 6a–c, the hydrophobic or hydrophilic nature of the surfaces evolved, particularly after the formation of the CR nanostructures in **NF100**. Initially, the PEDOT: PSS film exhibited a hydrophilic nature, with a WCA of 48.3 ± 3.45°. However, the uncoated **NF100** surface showed reduced hydrophilicity, with a WCA of 73.41 ± 0.04°. After an hour of incubation with the aqueous solution of PBA at 37 °C, the PBA-coated **NF100** surface became relatively more hydrophilic, with a WCA of 69.4 ± 0.15°. This increase in hydrophilicity is a crucial factor in enhancing the ability of the chip to maximize CTC capture yields and improve cell adhesion to the chip surface^18–20^ and plays an essential role in the operation of OECT devices. Figure 6d presents the XPS spectral analysis used to confirm the presence of PBA coating on the surface of CR-based NFAs through the π–π stacking interaction. The changes in the atomic percentages of C and O on **NF100** surfaces before and after PBA coating demonstrated increased PBA content on the CR surface. Particularly, the content of O 1s increased from 27.7% in uncoated **NF100** to 44.7% in PBA-coated **NF100**. This significant increase in the levels of O atoms, attributed to the carboxylic acid groups of PBA molecules, provides clear evidence of successful PBA coating on the CR surface.

**Figure 6:**
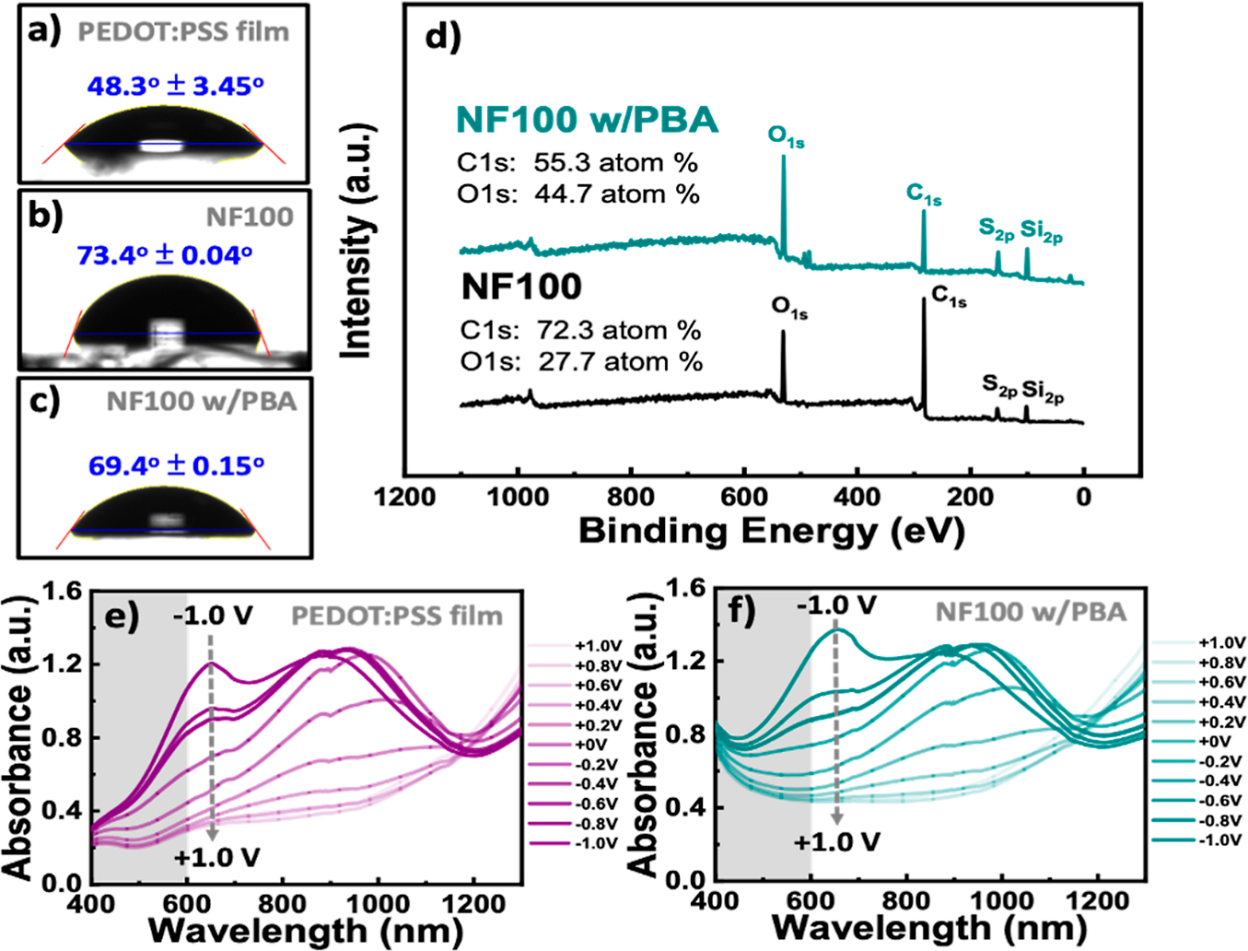
Wettability evaluation of (a) a pristine PEDOT:PSS (**P**) film, (b) **NF100**, and (c) **NF100** after surface modification with the PBA coatings. (d) XPS survey spectra of **NF100** before and after PBA-based surface modification. The UV–Vis–NIR absorbance spectra of the (e) pristine PEDOT:PSS (**P**) film and (f) **NF100** with PBA coating during electrochemical operation under a bias of −1.0 to +1.0 V vs Ag/AgCl electrodes in degassed 1× PBS.

Additional analysis based on UV–Vis absorbance under different applied potentials confirmed the effectiveness of PBA-coated **NF100** on the pristine PEDOT:PSS film, as shown in Figure 6e,f. The differences in absorbance behavior provide strong evidence supporting the presence of PBA-coated **NF100**, particularly enhanced absorbance peaks appearing in the 400–600 nm wavelength range compared with that in the pristine PEDOT:PSS film. For PBA-coated **NF100**, the absorbance in the 600–1300 nm range exhibited an optical response similar to that of the pristine PEDOT:PSS film when the applied potential was varied from −1.0 to +1.0 V. This consistency suggests that evenly PBA-coated **NF100** layer on the PEDOT:PSS film facilitates ionic-electron exchange in PBS during electrochemical operation.

To investigate long-term stability under electrochemical operation, transmittance at 650 nm and current density responses were analyzed for both pristine PEDOT: PSS film and PBA-coated **NF100** in PBS (Figure 7a–d,e–h, respectively) during cycles of applied potential switching between +0.6 V and −0.6 V. The pristine PEDOT:PSS film on ITO glass exhibited a greater difference in transmittance (increasing from an initial 34.7% to a final 36.0%) than that in the PBA-coated **NF100** (rising from an initial 14.7% to a final 18.6%) over 2500 s of device operation. This result aligns with the previous UV–Vis absorbance data (Figure 6e,f), as the absorbance peak of PBA-coated **NF100** is near 600 nm. Additionally, both PEDOT:PSS and PBA-coated **NF100** demonstrated excellent long-term stability in terms of optical and electrochemical properties, with minimal signal fluctuations over time. Notably, the PBA-coated **NF100** required an initial prerun period of approximately 250 s, which likely facilitated ionic electron exchange across the interfacial layers in the PBS buffer.

**Figure 7:**
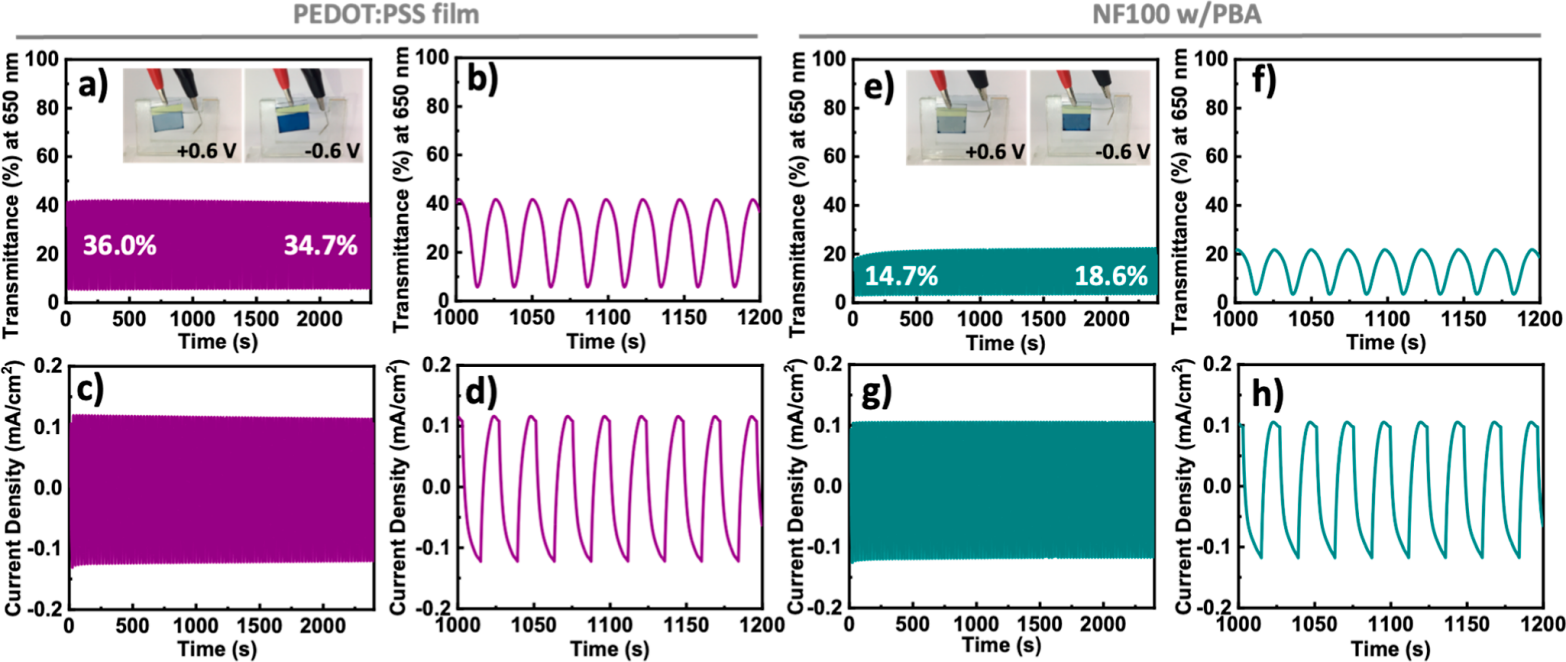
Long-time stability test of PEDOT:PSS (**P**) films and **NF100** with PBA coating during electrochemical operation under a bias switch between −0.6 V and +0.6 V vs Ag/AgCl electrodes, confirmed by the UV–Vis–NIR transmission (at 650 nm of wavelength) of (a,b) PEDOT:PSS (**P**) films, and (e,f) **NF100** with the PBA coating; confirmed by current responses of (c,d) PEDOT:PSS (**P**) films, and (g,h) **NF100** with the PBA coating.

In the present study, we observed significant *g*_m_ enhancement in OECTs by carefully tuning the deposited parameters of CR-based NFAs (**NF10**, **NF50**, **NF100**, and **NF300**) on the **P**-based OECT device (Figure 5). To investigate the effect of PBA on PEDOT:PSS films, additional experiments were conducted using **P**-based OECT devices with and without PBA modification (Figure S2). The results show that PBA incubation led to a slight decrease in transconductance and poorer on/off ratio during *I*_d_ *–V*_d_ measurements, as increasing the gate voltage failed to effectively turn off the drain current. Specifically, the on/off ratio dropped from 184.34 (pristine) to 7.96 after PBA modification. These findings suggest that PBA likely interacts selectively with CR-based NFAs via π–π interactions, rather than the PEDOT:PSS layer during the PBA coating step. However, in **NF10**- and **NF50**-based OECTs, where the nanofiber density and underlying thickness of NFAs are lower, PBA may penetrate into the PEDOT:PSS layer, leading to a reduction in transconductance.

To further evaluate the influence of the **NF100** coating onto ion transport kinetics and long-term stability for the PEDOT:PSS film, we performed a comparative analysis of drain current response (*I*_d_ *–*time) under repeated gate voltage switching over 10 day period (day 0, day 5, and day 10). The measurements involves 500 cycles of square *V*_g_ pulses (pulse amplitude: 0.1 V; pulse width: 1s; pulse period: 1s), with *V*_d_ maintained at – 0.2 V. On day 0 (Figure S3a–c), the **NF100**-based OECT exhibited a stable “spike and recovery” response throughout the 500 cycles, with the drain current decreasing slightly from the initial 100% to 98.9%, indicating negligible degradation. Similar responses were observed on day 5 and day 10, with the final drain current values of 98.3% and 99.0%, respectively. These measurements were conducted over three consecutive 500-cycle sessions using the same **NF100**-based OECT device, with stability assessed following a 5 day incubation period. These results confirm the robust and stable performance of the **NF100**-based OECT under prolonged dynamic operation, suggesting its potential to meet long-term requirements for clinical translation.

As reported in the previous reports,^49,50^ the potassium ions (K^+^) or other anions in PBS buffer or cell culture media can donate electrons to the CR π-system, forming charge–transfer complexes or facilitating anion intercalation. This doping process may enhance electrical conductivity and activate the full redox behavior of K_3_–CR complexes or intercalated species, which is advantageous for charge storage applications. We hypothesize that the **NF100** configuration represents an optimal balance between enhanced ionic–electronic coupling and preserved electron transport. Specifically, the moderate underlying thickness and nanofiber density generates a three-dimensional network that significantly shortens ion diffusion paths into the PEDOT:PSS layer, thereby boosting the mixed-capacitive charging responsible for high transconductance. At the same time, it maintains sufficient PEDOT:PSS connectivity to ensure effective charge carrier percolation—a condition that is compromised in thicker coatings such as **NF300** and **NF500**, where excessive CR thickness may hinder ion penetration and reduce performance. Furthermore, this **NF100** architecture may maximize the CCD measured via CV—an established predictor of transconductance for OECTs—by balancing charge storage and volumetric ion uptake.^5,22^ Taken together, these factors contribute to the pronounced transconductance peak observed in **NF100** devices, highlighting how nanofiber morphology control of CR-based NFAs can be strategically employed to optimize OECT amplification.

To demonstrate that the OECT device based on **NF100** can function as a NanoVelcro chip assay for the efficient capture and release of CTCs, MCF-7 (a human breast cancer cell line) and PC-9 (a lung adenocarcinoma cancer cell line) were used to evaluate its performance in targeted cell isolation and trigger cell release, driven by a CV sweeping operation (Figure 8a). Based on previous observations,^27^ a positively charged poly­(l-lysine)-*grafted*-poly­(ethylene glycol) (PLL-*g*-PEG–biotin) coating can readily be formed via electrostatic interactions on negatively charged PBA-modified CR-based NFAs containing carboxylic acid groups on their surfaces. Sequential coatings of streptavidin (SA) and biotinylated antihuman epithelial cell adhesion molecule (EpCAM) antibodies were applied through SA–biotin binding to enable specific cell isolation. Additionally, the 50% PEG side chains (without biotinylated functional groups) in PLL-*g*-PEG–biotin contributed to its antifouling properties, minimizing nonspecific binding. The optically transparent OECT channel based on **NF100** exhibited green fluorescence, demonstrating a synergistic effect in capturing most of the MCF-7 cells, which were stained with Hoechst 33342 (blue fluorescence), compared with that in a flat glass surface coated with the PLL-*g*-PEG–biotin/SA/biotinylated EpCAM antibody (Figure 8b,c). To further visualize the morphology of the MCF-7 cells captured on **NF100**, SEM was performed to confirm that the targeted cells exhibited a well-spread cellular morphology with extended pseudopodial structures, which enabled them to firmly grasp the nanofiber surface (Figure 8d).

**Figure 8:**
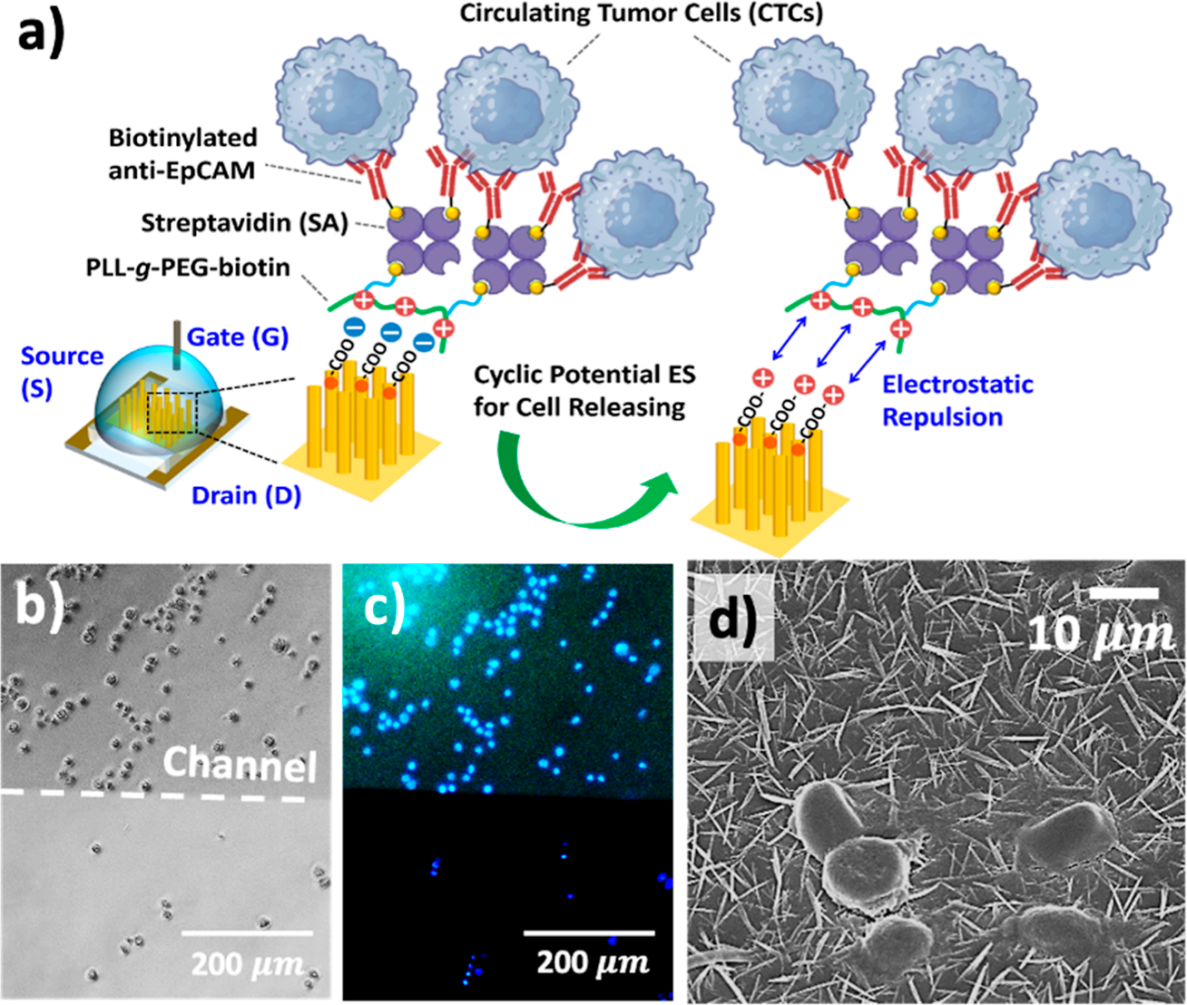
(a) Schematic illustration of the sequential coatings applied to the hybrid three-dimensional organic electrochemical transistor (3D-OECT) device for the isolation of circulating tumor cells (CTCs) and their electrically driven release via electrostatic repulsion. (b) Bright-field image and (c) corresponding fluorescence image of CTCs captured on a hybrid 3D-OECT channel layer, with cells stained using DAPI (4′,6-diamidino-2-phenylindole) for identification. (d) Top-view scanning electron microscopy image showing the CTCs captured on a hybrid 3D-OECT device.

To further quantify the cell capture and release performance of OECTs based on **NF100** with PLL-*g*-PEG–biotin/SA/biotinylated EpCAM antibody coatings, 200 μL of MCF-7 and PC-9 cell suspensions (at a concentration of 10^5^ cells per mL) were loaded onto devices featuring 10 mm-diameter PDMS reservoirs. After 60 min of cell capture, the devices were gently washed five times with 1× PBS. The captured cells were imaged and counted under different capture/release conditions using an inverted fluorescence microscope (CKX41; Olympus, Tokyo, Japan) (Figure 9a–f). The results demonstrated a more efficient capture of PC-9 cells, with an average cell density of approximately 400 cells mm^–2^ than that of MCF-7 cells at the average cell density of 225 cells mm^–2^. To determine the voltage required for effective cell release, we applied 20 cycles of the CV under various sweeping conditions (−0.8 to +0.5 V, 0 to +0.5 V, 0 to +0.8 V, and 0 to +1.0 V) at a scan rate of 100 mV s^–1^. The resulting decrease in cell density on CR-based NF devices (Figure 9a,d) demonstrated that a relatively high positive potential facilitated more efficient cell detachment through electrostatic interactions between the positively charged PEDOT:PSS and PLL-*g*-PEG–biotin coatings. Cell release was significantly more effective for PC-9, which achieved a total release efficiency of 99.94% when the sweeping voltage ranged from 0 to +1.0 V. This was confirmed by bright-field imaging after the capture and release processes for both cell lines (Figure 9b,c,e,f). Furthermore, the device demonstrated an isolation rate exceeding 90% for breast MCF-7 cancer cells spiked into THP-1 cell solutions (10^6^ cells mL^–1^), with minimal nonspecific binding. Moreover, over 80% of captured CTCs were released from the CR-based NFAs during CV sweeps ranging from 0 to +1.0 V in PBS.

**Figure 9:**
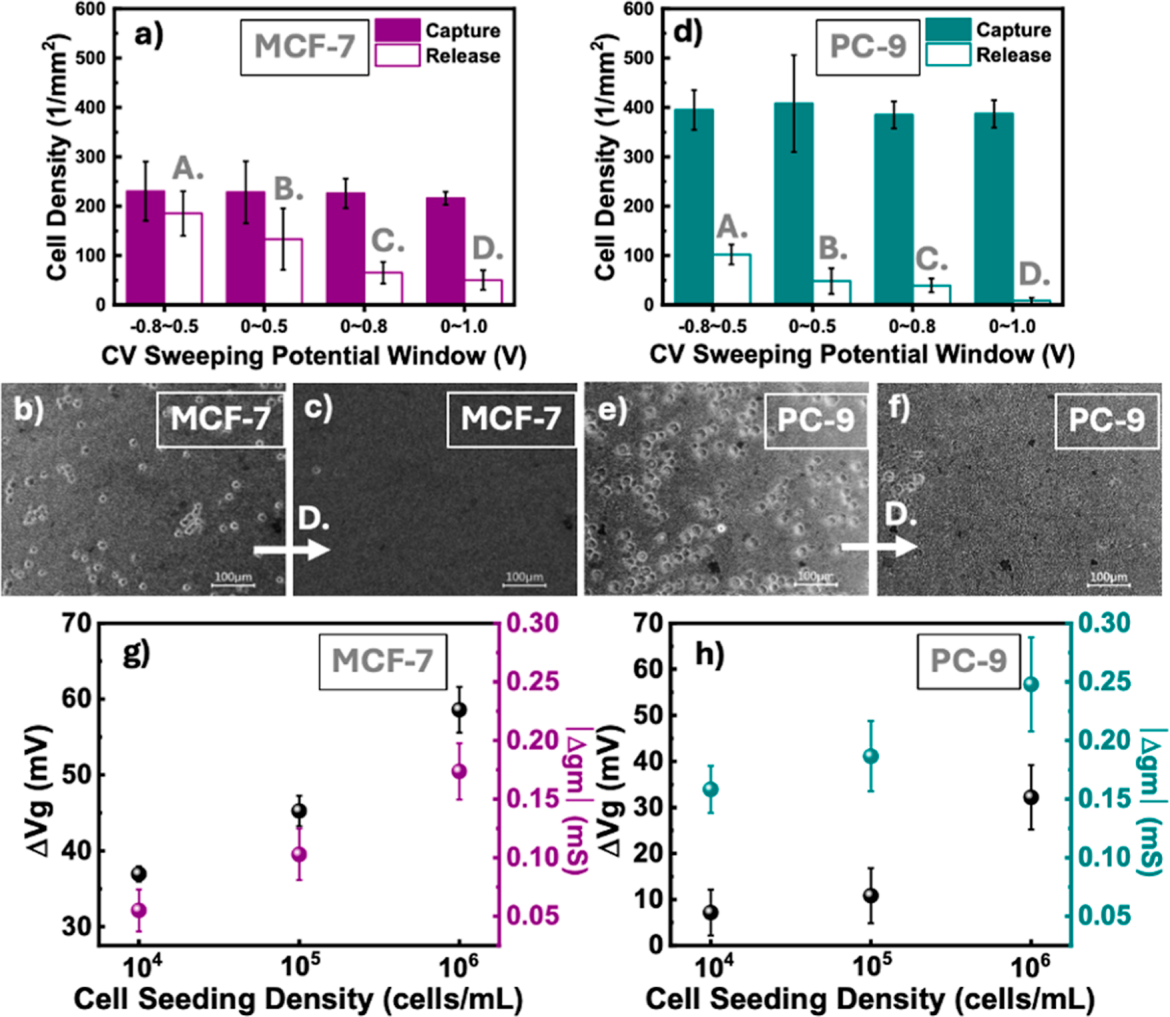
Evaluation of circulating tumor cell (CTC) isolation and release performance. (a) Graph depicting the cell densities of captured and released MCF-7 cells under different potential sweeps. (b,c) Bright-field images showing MCF-7 cells captured and released after 20 cycles of 0–1 V potential sweeps, respectively. (d) Graph depicting the cell densities of captured and released PC9 cells under different potential sweeps. (e,f) Bright-field images showing PC9 cells captured and released, respectively, at 20 cycles of the potential sweeps of 0–1 V. (g) Changes in the gate voltage and transconductance of organic electrochemical transistor (OECT) devices for biosensing the MCF-7 cell density difference. (h) Changes in the gate voltage and transconductance of OECT devices for biosensing differences in PC9 cell density.

Following these observations, we studied the relationship between cell seeding density (10^4^, 10^5^, and 10^6^ cells mL^–1^) and the electrical signal response of the device, including changes in the gate voltage (Δ*V*_g_) and transconductance (Δ*g*_m_) of MCF-7 and PC-9 cells on OECTs based on **NF100** with PLL-*g*-PEG–biotin/SA/biotinylated EpCAM antibody coatings (Figure 9g,h, respectively). This analysis provided detailed insights into the impact of the number of captured cells on the electrical characteristics of the OECTs. Particularly, higher cell densities result in a greater load on the devices, and as the number of cells increases, cumulative capacitive effects likely alter the gate voltage and transconductance responses, requiring larger Δ*V*_g_ and Δ*g*_m_. For different cell lines, MCF-7 cells showed larger average Δ*V*_g_ values of 36.9, 45.2, and 58.6 mV for seeding densities of 10^4^, 10^5^, and 10^6^ cells mL^–1^, respectively, than those of PC-9 cells at 7.2, 10.8, and 32.2 mV, respectively (Figure 9g). By contrast, PC-9 cells exhibited larger average Δ*g*_m_ values of 0.16, 0.19, and 0.25 mS at the seeding densities of 10^4^, 10^5^, and 10^6^ cells mL^–1^, respectively (Figure 9h), whereas MCF-7 showed smaller values of 0.06, 0.10, and 0.17 mS, respectively (Figure 9g). These OECT results demonstrate that both the captured cell density and cell type impact the levels of Δ*V*_g_ and Δ*g*_m_ changes, providing a basis for cell counting and cell type identification.

To further demonstrate the capability of the **NF100**-based OECT for monitoring ΔVg values under various conditions—including baseline (precapture), post-CTC capture, and postrelease—we evaluated the device under four defined conditions: **T0** condition: **NF100**-based OECT with a PBA coating, **T1** condition: T0 modified with PLL-*g*-PEG–biotin/SA/biotinylated EpCAM antibody, **T2** condition: T1 following MCF-7 cell capture (seeding density = 10^5^ cells/mL), and **T3** condition: T2 after 20 cycles of CV sweeps (0 to +1.0 V in PBS) to induce cell release (Figure S4). The results confirm that the **NF100**-based OECT can effectively monitor each modification and cell-handling step, exhibiting a Δ*V*_g_ shift of 47.7 mV from **T0** to **T1**. Upon MCF-7 cell capture (**T2**), a further Δ*V*_g_ increase of 118.2 mV was observed relative to **T1**. Following electrochemical release (**T3**), the Δ*V*_g_ decreased to 11.6 mV, approaching the baseline level (**T0**), thereby confirming successful cell release and surface regeneration (Figure S4e,f). Additionally, a comparison table with corresponding references has been provided (Table S1) to facilitate a direct quantitative comparison with existing CTC-capture platforms. Our **NF100**-based OECT exhibits a high capture efficiency (>90%) comparable to leading technologies. Moreover, it uniquely integrates immunofluorescence imaging and electrical signal monitoring to evaluate CTC capture and release performance, and enables controllable cell release via CV operation.

A parallel process using **NF100**-based OECTs with PLL/collagen coatings was performed for immortalized bone marrow stem cell (IBMSC) line culture, followed by ES treatment to enhance EV secretion (Figure 10). As shown in Figure 10a,b, PLL and collagen were sequentially deposited onto the PBA-coated CR-based NFAs via electrostatic interactions, forming a functional OBEI for cell culture and pulsed ES treatments (Figure 10c,d, respectively). The ES protocol was applied using three different voltage levels (5, 10, and 20 V) at a frequency of 0.5 Hz, with a pulse duration of 5 ms over 72 h. Figure 10e presents the EV release profiles, obtained by the nanoparticle tracking analysis (NTA) method. The data show the EV concentrations as a function of particle size under different voltage conditions (control, 5, 10, and 20 V). These results indicate that increasing the ES voltage significantly enhances EV secretion, demonstrating the effectiveness of our **NF100**-based OECT platform. Notably, 20 V of ES yielded the highest EV concentration. The size distribution of the released EVs predominantly falls within 50–200 nm, a characteristic of biologically relevant EVs. This confirmed that ES effectively promoted EV release from IBMSCs cultured on the chip. The observed behavior suggests that ES directly impacts cellular activity, potentially through electromechanical stimulation or membrane depolarization.^51,52^

**Figure 10:**
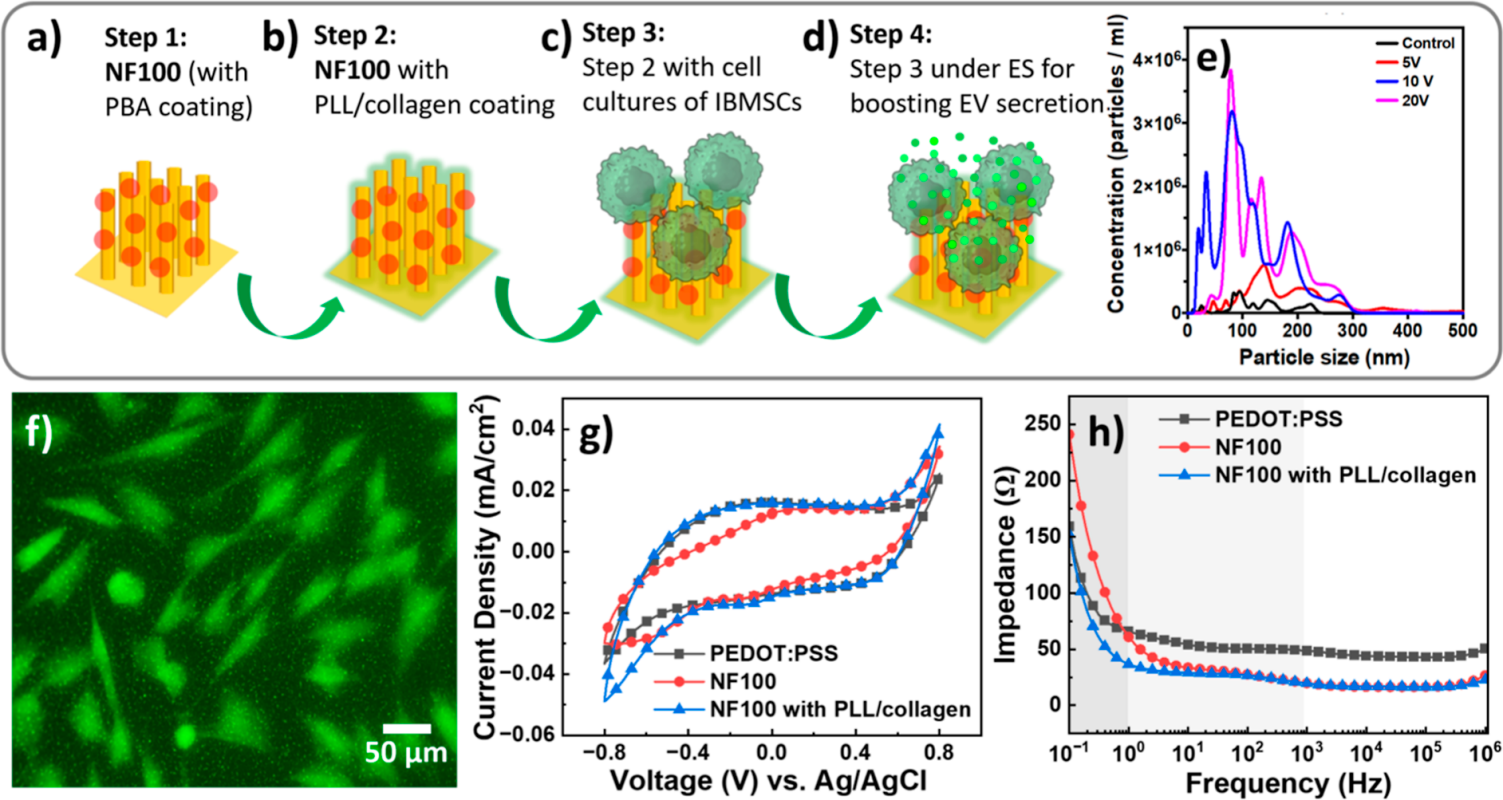
(a–d) Schematic illustration of enhanced EV production during ES operation using a 3D-OECT device. (e) Particle counts of EVs derived from IBMSCs under different ES conditions, with applied voltages of 0 (control), 5, 10, and 20 V. (f) Fluorescence image from the live/dead assay showing of IBMSCs on **NF100** device with the PLL/collagen coating. (g) CV curves and (h) EIS responses across a frequency range of 0.1 to 10^4^ Hz for ITO electrodes with various coatings, including PEDOT:PSS, **NF100**, and **NF100** with the PLL/collagen coating. The potential was swept from −0.8 to +0.8 V vs Ag/AgCl electrodes at a scan rate of 100 mV s^–1^, with CCDs of 0.351, 0.302, and 0.402 mC cm^–2^, respectively.

To evaluate the cell adhesion and biocompatibility of **NF100**-based OECT devices after ES treatment, fluorescence imaging of IBMSCs stained with a live/dead assay was performed. The results confirmed that the most of the live cells (green) efficiently adhered to CR-based NFAs with PLL/collagen coatings, demonstrating high cell viability under an ES of 10 V. This suggests that these coatings have the potential to enhance stem cell-derived EV secretion (Figure 10f). To assess the charge injection and charge transport performance of 3D-OBEIs (ITO, PEDOT: PSS, **NF100**, and **NF100** with the PLL/collagen coating) during electrochemical operation, CV and EIS were performed in 1× PBS (Figure 10g,h, respectively). Regarding the CV analysis results, CCDs estimated for PEDOT: PSS, **NF100**, and **NF100** with PLL/collagen coating were 0.351, 0.302, and 0.402 mC cm^–2^, respectively, indicating that **NF100** with PLL/collagen coating had the highest charge injection performance under ES (Figure 10g). As shown in Figure 10h, the EIS results revealed that **NF100** with PLL/collagen coating exhibited (1) lower impedance in the low-frequency region (mHz to Hz), indicating enhanced ion–electron coupling efficiency; (2) lower impedance in the medium-frequency region (Hz to kHz), suggesting reduced interfacial charge resistance; and (3) lower impedance in the high-frequency region (kHz to MHz), reflecting faster charge carrier movement and higher electronic conductivity of the active material than those in ITO, PEDOT:PSS, and **NF100**. These findings highlight the superior electrochemical performance of **NF100** with a PLL/collagen coating, making it a promising candidate for OBEI to provide efficient ES for enhanced EV secretion applications.

Experiments to enhance EV production were conducted using MCF-7, a human breast cancer cell line, and IBMSC cultures on 3D-OECT devices based on **NF100** with a PLL/collagen coating. ES was applied at three different voltage levels (5, 10, and 20 V) at a frequency of 0.5 Hz, with a pulse duration of 5 ms over 72 h (Figure 11). For both cell types, cell viability decreased with increasing voltage *V*_d_, indicating a certain degree of cellular stress at relatively high voltages under ES conditions. However, EV concentration (measured in particles per milliliter using NTA) increased significantly with voltage, peaking at 20 V. This suggests that despite reduced viability at relatively high voltages, the cells continued to release substantial quantities of EVs, potentially as a stress response or because of apoptosis. Notably, IBMSCs produced significantly higher amounts of EVs before and after ES than those in MCF-7 cells (Figure 11a,b). This can be attributed to the intrinsic properties of IBMSCs, which are involved in tissue repair, cell signaling, and immune modulation. Bright-field images were captured on the interdigital **NF100**-based OECT devices before and after ES for a detailed investigation of the cellular response (Figure 11c,d). The observed MCF-7 and IBMSC cell attachment areas are located on the **NF100**-coated PEDOT:PSS/ITO source electrodes, the **NF100**-coated PEDOT:PSS channel region, and the **NF100**-coated PEDOT:PSS/ITO drain electrodes. Furthermore, fluorescence imaging of MCF-7 cells on the **NF100**-based OECT devices was performed using Hoechst 33,342 (blue) to stain nuclei and Phalloidin (green) to stain the cytoskeleton (Figure S5). The images reveal robust adhesion of MCF-7 cells on the CR-based NFAs (green), with well-spread cell bodies, intact nuclei, and extensive cytoplasmic extensions along the nanofiber scaffold, indicating healthy morphology and strong cell–substrate interactions. These images showed that most cells remained intact on the 3D-OECT device surface before ES, indicating adequate structural integrity and cell adhesion.

**Figure 11:**
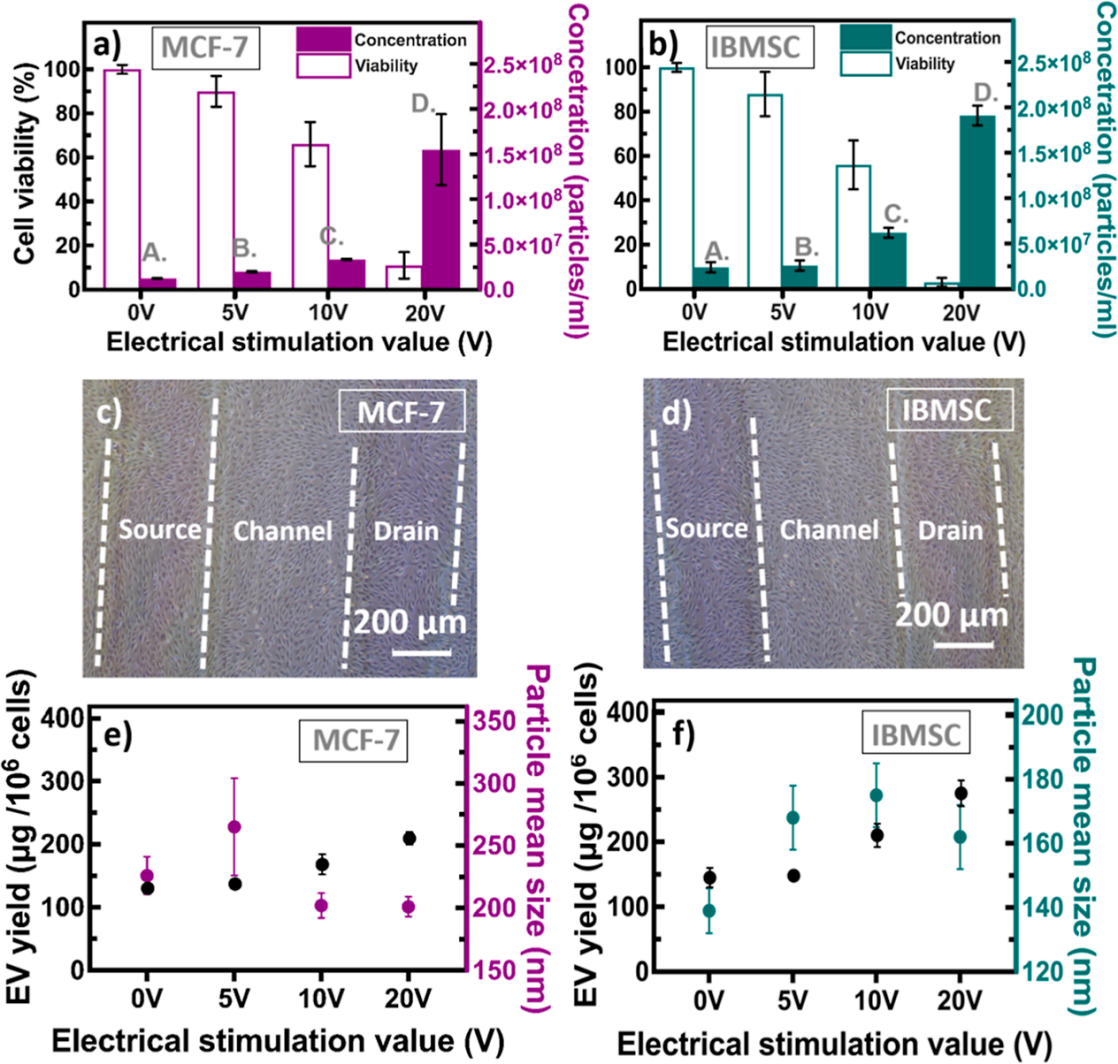
Enhancement of extracellular vesicle (EV) production using interdigital 3D-OECT devices under different electrical stimulation (ES) conditions, with the applied voltages of 0 (control) and 5, 10, and 20 V. (a) Cell viabilities and EV concentrations derived from MCF-7 cells. (b) Cell viabilities and EV concentrations derived from IBMSCs. Bright-field images of (c) MCF-7 and (d) IBMSCs on devices before ES. The yields and particle size distributions of EVs derived from (e) MCF-7 cells and (f) IBMSCs.

Additionally, the relationship between EV yield (correlated with protein yield) and EV size under different ES conditions was examined in both MCF-7 cells and IBMSCs (Figure 11e,f). The highest EV production was observed at 20 V, with a 12.4-fold increase in MCF-7 cells and an 8.0-fold increase in IBMSCs compared with that at 0 V (control) without significantly altering the EV size or requiring additional cellular modifications. EV-protein yield, measured in micrograms per million cells using bicinchoninic acid assay, increased with voltage, suggesting that ES enhances EV release efficiency. By contrast, EV particle size remained relatively stable across all conditions, indicating that MCF-7 cells and IBMSCs predominantly produce smaller EVs under stress than under nonstress conditions. This may be attributed to changes in the membrane dynamics or vesicle biogenesis pathways in response to ES. Although the precise mechanisms by which ES enhances EV secretion remain to be fully elucidated, we speculate a Ca^2+^–centric intracellular signaling pathway, based on findings from related studies.^53,54^ First, low-level electrical fields have been reported to induce transient elevations in cytosolic Ca^2+^, which subsequently activate Rho GTPases via HSP90 and protein kinase C, leading to the phosphorylation of Rab-10—a key regulator of vesicle trafficking. These processes collectively promote exosome biogenesis and release.^53^ Second, studies on cellular nanoporation have shown that localized membrane perturbations and heating rapidly increase intracellular Ca^2+^ levels and heat shock protein expression, triggering the p53–TSAP6 axis, which further drives exosome formation and secretion.^54^ Under our ES conditions (5–20 V pulses at 0.5 Hz), we similarly anticipate Ca^2+^ influx through voltage-gated or mechanosensitive channels, resulting in HSP upregulation, activation of Rho and Rab family proteins, and engagement of the p53–TSAP6 pathway. These converging mechanisms likely synergize to modulate the endosomal sorting complexes required for transport machinery, facilitating membrane budding into multivesicular bodies and ultimately enhancing EV release.

Overall, the 10 V ES condition using our 3D-OECT device resulted in an approximately 2.7-fold increase in EV production compared with that in the 0 V control while maintaining a balance between cell viability (MCF-7:66 ± 10%; IBMSC: 56 ± 11%) and EV-protein yield (MCF-7:168 ± 16 μg/10^6^ cells; IBMSC: 210 ± 18 μg/10^6^ cells). To better contextualize the EV enhancement achieved by our 3D-OECT platform, we compared its performance against a range of established ES methods (Table S2). Our device achieved a remarkable 12.4-fold increase in EV secretion across multiple cell types, substantially exceeding the enhancements reported for other techniques, including a 1.2-fold increase with contractile-workload stimulation, 1.7-fold with low-level direct current, and 2-fold with low-frequency electrical acupuncture. Only cellular nanoporation, which induces membrane poration and localized heating, demonstrated a higher enhancement of approximately 50-fold. Aside from nanoporation, our NF100-based OECT outperforms all other ES strategies, positioning it among the most effective platforms for EV production. We anticipate that further optimization of ES parameters and device design will yield even greater performance shortly. Additionally, further biological studies must investigate the underlying mechanisms, including the roles of membrane proteins and gene expression, to fully realize the potential of the 3D-OECT device for EV-based applications.

## 4 Conclusion

In this study, we developed a versatile 3D-OECT platform integrating CR-based NFAs on a PEDOT:PSS active-layer channel, enabling both cancer cell capture and electrically driven cell release for the targeted liquid biopsy enrichment process, as well as ES treatment for enhanced EV production. Our findings demonstrate that this platform provides two major functions: (1) cancer cell capture/release and biosensing functions, and (2) EV production enhancement. Regarding the cancer cell capture/release and biosensing functions, the **NF100**-based OECTs, functionalized with PBA via π–π stacking with CR, exhibited enhanced transconductance compared with that of the pristine PEDOT:PSS-based OECTs. This functionalization improves the CCD, lowers the electrochemical impedance, enhances the stability, and increases the charge-injection capacity. For the NanoVelcro chip assay, sequential surface modifications with the PLL-*g*-PEG–biotin/SA/biotinylated EpCAM antibody further optimize CTC capture performance. Additionally, our 3D-OECT device enables efficient, electrically driven cell release through CV sweeping (0 to +1.0 V) in PBS, whereas biosensing captures the cell density across different seeding densities (10^4^, 10^5^, and 10^6^ cells mL^–1^) and allows monitoring of both cell capture and release through corresponding Δ*V*_g_ shifts. Regarding the EV production enhancement function, the **NF100**-based OECTs, further modified with PLL/collagen, promoted cell adhesion and biocompatibility, supporting ES treatment for boosting EV production without additional biochemical additives in the culture medium. The optimization of ES conditions (0–20 V) in FBS-free medium revealed a peak EV yield at 20 V, with a 12.4-fold increase in MCF-7 cells and an 8.0-fold increase in IBMSCs compared with those at 0 V. At 10 V, EV production increased 2.7-fold while maintaining cell viability (MCF-7: approximately 66%; IBMSC: approximately 56%) and high EV-protein yield (MCF-7: approximately 168 μg/10^6^ cells; IBMSC: approximately 210 μg/10^6^ cells). This proof-of-concept 3D-OECT device, which leverages CR-based NFAs, bridges the gap between OECTs and biological applications. Our approach holds promise for advancing next-generation bioelectronic therapies by broadening the device operation mechanisms.

## Supplementary Material



## Data Availability

The data that has been used is confidential.

## Notes

Morphological and electrochemical characterizations of the CR-based NFAs. CV and EIS of PEDOT:PSS film with CR-based NFAs. Surface modification process of 3D-OECT devices. Cell studies and their applications for cell capture and release, and enhanced EV production experiments on 3D-OECT devices. Photograph of the NF100-based OECT device; Output curves (*I*_d_ *–V*_d_) and transconductance curves (*g*_m_–*V*_g_) of P-OECTs before and after PBA surface modification; long-term (*I*_d_–time) stability assessment of NF100-based OECT; comparative analysis of CTC isolation and detection technologies; comparison of EV production performance across various ES technologies (PDF)
