# Supplementary material for: Versatile Organic Electrochemical Transistors with Self-Assembled Coronene Nanofiber Arrays for the Isolation and Detection of Circulating Tumor Cells and Enhanced Secretion of Extracellular Vesicles
Source: ACS Appl Mater Interfaces. 2025 May 30;17(23):33592–605. doi: 10.1021/acsami.5c05442 (PMC12163892; doi:10.1021/acsami.5c05442)
Supplement: Supplementary file 1 [file am5c05442_si_001.pdf]

Supporting Information

Versatile Organic Electrochemical Transistors with Self-Assembled Coronene Nanofiber Arrays for the Isolation and Detection of Circulating Tumor Cells and Enhanced Secretion of Extracellular Vesicles

Edgar Daniel Quiñones,[a,b,#] Jiashing Yu,[b,c,#] Rou-Zhen Liu,[d] Yi-Shiuan Li,[a] Yu-Chuan Lu,[e,f] Yu-Sheng Hsiao[a,g*]

[a]Department of Materials Science and Engineering, National Taiwan University of Science and Technology, Taipei 106335, Taiwan; E-mail: yshsiao@mail.ntust.edu.tw

[b]Taiwan International Graduate Program, Sustainable Chemical Science & Technology, Academia Sinica, Institute of Chemistry, Taipei 115, Taiwan

[c]Department of Chemical Engineering, National Taiwan University, Taipei 10617, Taiwan

[d]Department of Materials Engineering, Ming Chi University of Technology, New Taipei City 24301, Taiwan

[e]Department of Surgical Oncology, National Taiwan University Cancer Center, National Taiwan University College of Medicine, Taipei 10672, Taiwan

[f]Department of Urology, National Taiwan University Hospital, Taipei 100225, Taiwan

[g]Energy and Sustainability Tech Graduate Institute, National Taiwan University of Science and Technology, Taipei 106335, Taiwan

[*]Corresponding author at:

Prof. Yu-Sheng Hsiao: Department of Materials Science and Engineering, National Taiwan University of Science and Technology, Taipei 10607, Taiwan; Energy and Sustainability Tech Graduate Institute, National Taiwan University of Science and Technology, Taipei 10607, Taiwan; E-mail: yshsiao@mail.ntust.edu.tw

# Edgar Daniel Quiñones and Jiashing Yu contributed equally to this work.

Morphological and electrochemical characterizations

To analyze and catalog the morphological changes of the CR-based NFAs, field emission-scanning electron microscopy (FE-SEM, FEI Nova NanoSEM 200; accelerating voltage: 10 kV) was used to record the top and cross-sectional views of samples. The CR-based NFAs were examined for statistically significant variations in growth length and diameter across different conditions. Subsequently, the crystalline structures of CR powder and CR-based NFAs were characterized using grazing-incidence X-ray diffraction (GIXRD; Philips PANalytical X'Pert PRO MRD apparatus; Cu Kα radiation). The incident X-ray beam angle was fixed at 5° above the critical angle to ensure accurate measurements. Contact angles (CAs) were determined using the geometric mean approximation with DI water to evaluate surface wettability. The chemical configurations of the samples were analyzed using X-ray photoelectron spectroscopy (XPS; PHI5000 VersaProbe apparatus), employing a monochromatic Al Kα X-ray source (1486.6 eV) for excitation.

Cyclic voltammetry (CV) and electrochemical impedance spectroscopy (EIS) were performed using a PGSTAT204 potentiostat/galvanostat (Autolab, Eco Chemie, Netherlands) equipped with a frequency response analysis (FRA) module. The experiments utilized a three-electrode setup within a glass cell, with the sample serving as the working electrode, a platinum wire as the counter electrode, and an Ag/AgCl electrode (RE-1B; ALS Co. Ltd, Japan) as the reference electrode. For CV measurements, 1× phosphate-buffered saline (PBS) was used as an electrolyte, and the potential was swept from −0.8 to +0.8 V versus Ag/AgCl at a scan rate of 100 mV s^{-1}. For EIS, 1× PBS was also employed as the electrolyte. An alternating current (AC) voltage with an amplitude of 5 mV was applied to evaluate EIS results across a frequency range of 0.1 to 10 kHz, measured at open circuit potential.

Surface modification of 3D-OECT devices

All active-layer channels of the 3D-OECT devices were initially functionalized with a modified solution of 2mM 1-pyrenebutyric acid (PBA, 97%, Sigma- Aldrich) in 0.1 M sodium tetraborate (STB) buffer (pH 9.2) for 1 h on the CR-based NFAs. After the PBA functionalization, the devices were thoroughly washed with sterilized DI water to remove any excess non-adsorbed PBA. The 3D-OECT devices were then separated for further modifications tailored to specific applications in CTC isolation and enhanced EV production.

For the CTC isolation and release experiments, a solution of PLL(20)-*g*-[3.5]-PEG(2)/PEG(3.4)-biotin(50%) (PLL-*g*-PEG-biotin, SuSoS AG, Switzerland) was prepared at a concentration of 100 mg mL^{-1} in 10 mM 4-(2- hydroxyethyl)-1-piperazineethanesulfonic acid (HEPES) buffer (pH 7.4). This solution was used to modify the PBA-functionalized 3D-OECT for 1 h. The devices were incubated with streptavidin (SA, Invitrogen, USA) (10 mg mL^{-1} in 1× PBS) for 1 h at room temperature. Next, a biotinylated anti-human epithelial cell adhesion molecule (EpCAM)/TROP1 antibody (R&D systems, USA) solution [10 mg mL^{-1} in 1× PBS containing 0.1% bovine serum albumin (BSA) and 0.09% NaN_3; 25 mL] was applied to the device and incubated for 1 h at room temperature. Finally, the devices were washed multiple times with 1× PBS and immersed in 1× PBS for 1 h before conducting the cell experiments.

Cell studies

The lung adenocarcinoma cancer cell line (PC-9) was kindly provided by Prof. Sung-Liang Yu (Department of Clinical Laboratory Sciences and Medical Biotechnology, College of Medicine, National Taiwan University, Taiwan). The breast cancer cell line (MCF-7), human monocytic cell line (THP-1), and immortalized bone marrow cell line (IBMSC) were obtained from the Bioresource Collection and Research Center (BCRC, Taiwan). The growth medium for the MCF-7 cell line was Dulbecco's Modified Eagle's Medium (DMEM), while the IBMSC cells were cultured in α-MEM low glucose medium, and both PC-9 and THP-1 cells were maintained in RPMI 1640. All media were supplemented with 10% fetal bovine serum (FBS, Hyclone) and

PSA (100 mg mL^{-1}). Cell cultures were incubated at 37 ºC in a humidified atmosphere with 5% CO_2 using a cell incubator (Thermo Scientific, Thermo 370). DMEM and RPMI 1640 medium were sourced from Invitrogen, and HEPES buffer was obtained from Life Technologies.

3D-OECT devices for cell capture and release experiments

After removing the PBS buffer from the 3D-OECT device with the anti-human epithelial cell adhesion molecule (EpCAM) antibody modification, a cell suspension with a concentration of 10^5 cells mL^{-1} (200 μL) was loaded onto the device. The cells were incubated for 1 h at (37°C, 5% CO_2). Following incubation, the device was carefully washed five times with 1× PBS. A fluorescence microscope (CKX41, Olympus) was used for bioimaging and cell counting. Cell counts, dilution, and viabilities were determined using an automated cell counter (Luna^{TM} automated cell counter, Logos Biosystems, Korea). Cell viability experiments were conducted in triplicate. For cell release experiments under ES operation, a standard three-electrode setup was employed with a working electrode. Cyclic potential ES was applied using four different voltage sweeps: from −0.8 to +0.5 V, from 0 to +0.5 V, from 0 to +0.8 V, and from 0 to +1.0 V, at a scan rate of 100 mV s^{-1} for 20 cycles in 1× PBS. All experiments were performed at least three times. Cell capture and release data are expressed as mean ± standard deviation (SD) for n=900 cells and showed consistent results.

3D-OECT devices for enhanced EV production experiments

After removing the PBS buffer from the 3D-OECT device with collagen modification, the standard medium, along with a cell suspension at a concentration of 1× 10^6 cells mL^{-1} (1000 μL), was seeded onto the device. The cells were incubated for 24 h (37°C, 5% CO_2). Following incubation, the device was carefully washed three times with 1× PBS and subsequently incubated with an FBS-free medium. ES experiments were conducted using a standard two-electrode setup, employing the source and drain electrodes. Pulsed ES was applied with three voltage levels (5 V, 10 V, and 20 V) at a frequency of 0.5 Hz, with a pulse period of 5 ms for 72 h. All experiments

were repeated at least three times, with data expressed as mean ± SD, and provided consistent results. After the ES treatment, the media from different ES conditions were collected from the devices. A total cell count of 1×10^6 cells was registered for each device. The collected media were centrifuged at 500×g for 10 min to remove large cell debris. The supernatant was further collected and centrifuged again at 1000×g for 15 min to eliminate larger apoptotic vesicles. The resulting supernatant was then transferred into a Macrosep Centrifugal Filter Unit (MWCO =100 kDa; Pall Life Sciences) and centrifuged at 5000×g for 30 min. The concentrated medium was then processed using a Microsep Centrifugal Filter Unit (MWCO =100 kDa; Pall Life Sciences) and centrifuged at 7500×g for 10 min to obtain the final concentrated EV-rich medium. For the isolation of EVs *via* size exclusion chromatography (SEC), the pre-concentrated EV-rich medium was passed through a commercially available SEC column (qEV Original/70 nm; Izon Science, New Zealand) with a separation range of 50–200 nm. Fractions were collected in 0.4 mL volumes using an automatic fraction collector (AFC-V1, Izon Science, New Zealand), yielding a total of seven fractions. The desired fractions were recovered and stored for future use. Potassium-deficient PBS was used as the elution buffer in the SEC column. The protein concentration of the collected EVs was determined by using a Micro-bicinchoninic acid (BCA) Protein Assay Kit (Pierce Biotechnology, Rockford, IL, USA), following the manufacturer's instructions. The absorbance of each sample at 562 nm was measured using a microplate reader (TECAN Infinite M200 PRO, Austria).

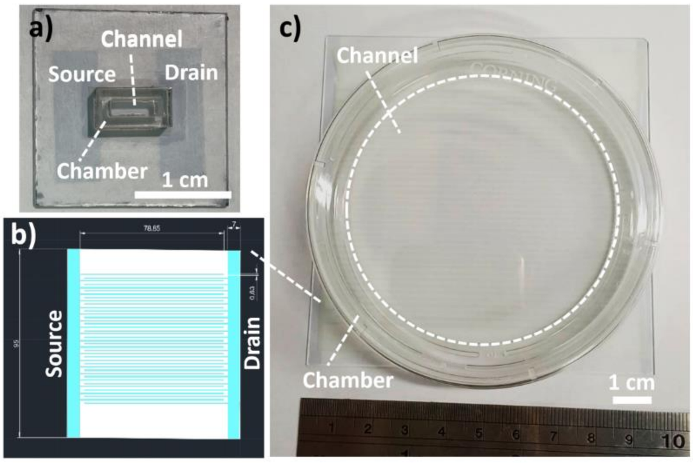

Figure S1. (a) Photograph of the **NF100**-based OECT device used for monitoring ΔVg shifts during the surface modification and cell capture/release processes. (b) AutoCAD layout and (c) corresponding photograph of the **NF100**-based OECT device integrated with a commercial 10 cm polystyrene cell culture dish for EV production.

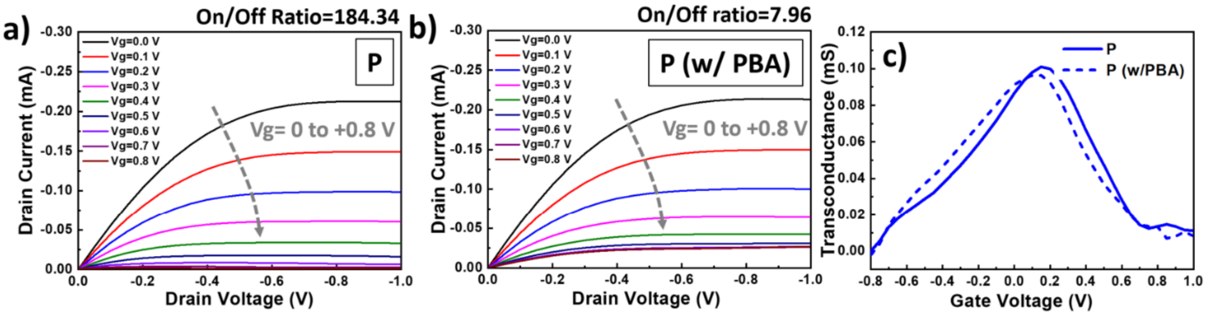

Figure S2. Output curves (I_d–V_d) of OECTs based on (a) a pristine PEDOT: PSS film (**P**) and (b) **P** after surface modification with the PBA coating. (c) Representative transconductance curves (g_m–V_g) for the **P**-OECT before and after PBA surface modification, measured at a constant V_d of −0.1 V.

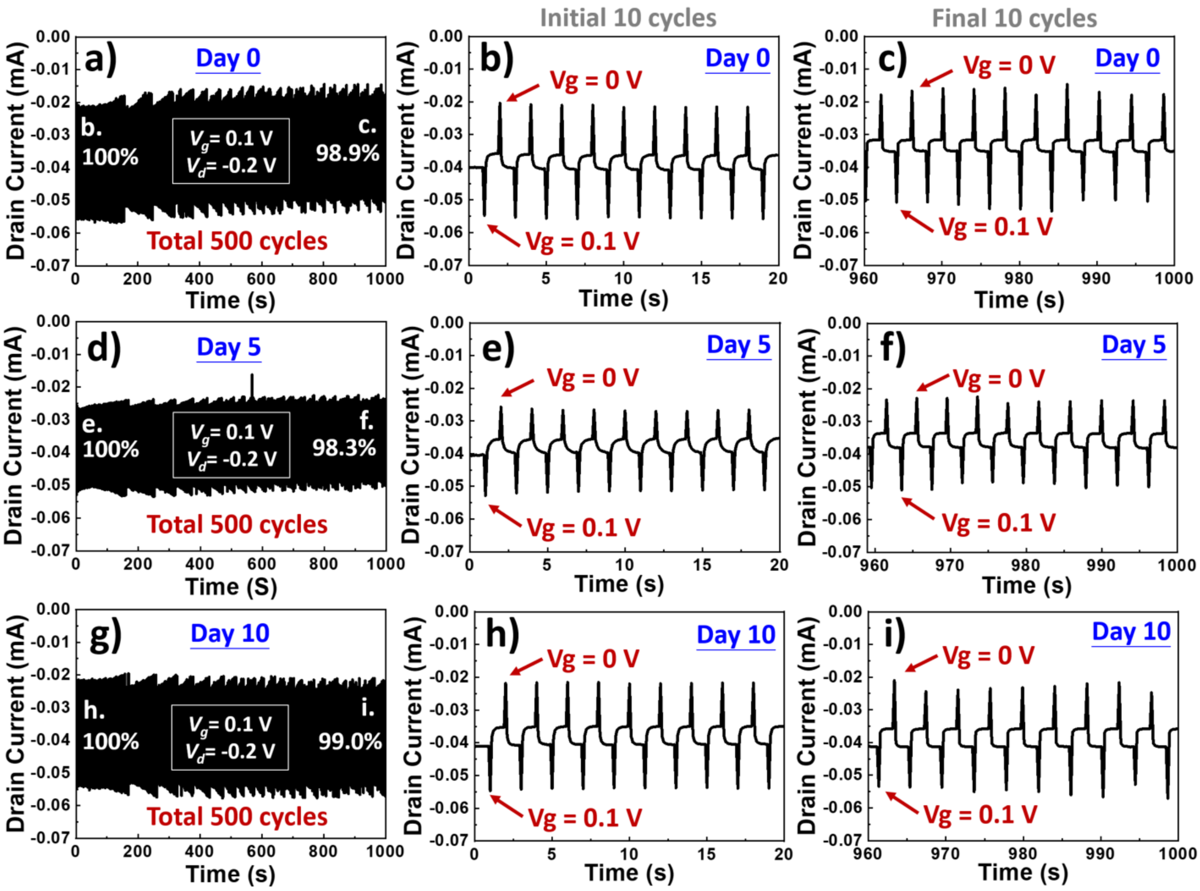

Figure S3. Long-term (I_d-*Time*) stability assessment of **NF100**-based OECT in PBS buffer on (a–c) Day 0, (d–f) Day 5, and (d–f) Day 10. The transient responses were recorded over 500 cycles of square V_g pulses (pulse amplitude: 0.1 V; pulse width: 1 s; pulse period: 1 s), with V_d maintained at −0.2 V, initiated at Day 0 of device operation. The initial 10-cycle results were shown on (b) Day 0, (e) Day 5, and (h) Day 10, while the final 10-cycle results were shown on (c) Day 0, (f) Day 5, and (i) Day 10.

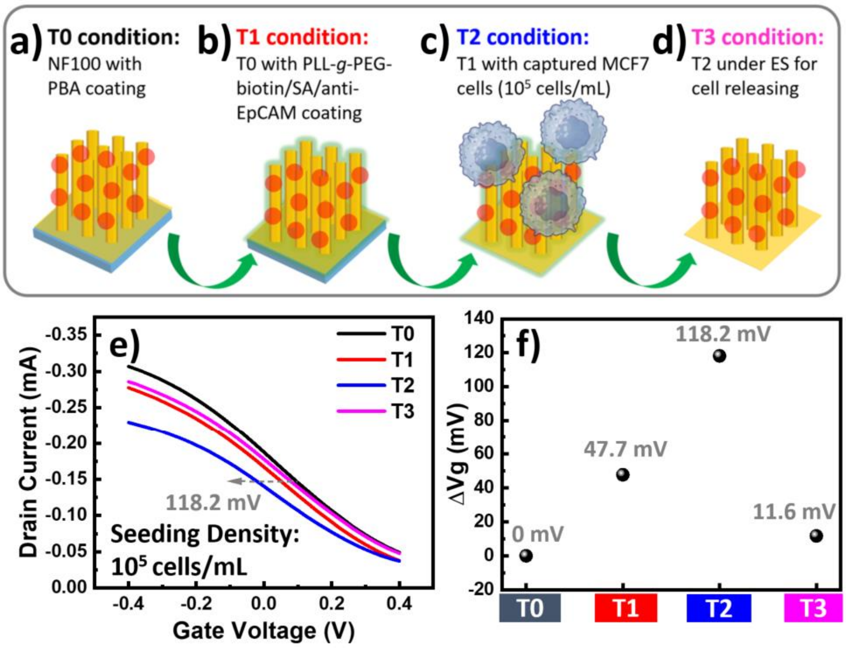

Figure S4. (a–d) Schematic illustrations of the **NF100**-based OECT used to monitor the ΔVg shifts under various conditions: **T0** (PBA-coated device), **T1** (after PLL-g-PEG–biotin/SA/biotinylated EpCAM antibody modification), **T2** (after MCF-7 cell capture), and **T3** (after CV-driven cell release). (e) Transfer curves (I_d–V_g) of the device under each condition, and their (f) corresponding ΔVg shifts.

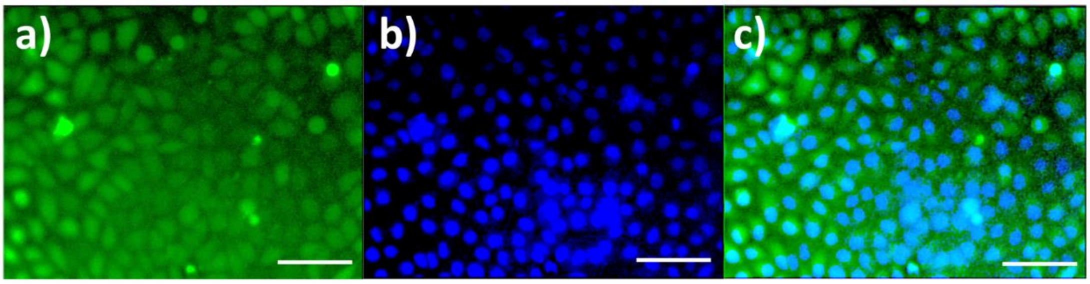

Figure S5. Fluorescence images of MCF-7 cells cultured on **NF100**-based OECT devices for EV production. (a) Green fluorescence showing cytoskeleton staining with Phalloidin, with CR-NFAs also visible in green. (b) Blue fluorescence showing nuclei staining with Hoechst 33342. (c) Merged fluorescence image. Scale bar: 100 μm.

Table S1. Comparative analysis of CTC isolation and detection technologies.

Rare Cell Isolation Platform/Method	Capture Efficiency (%)	Detection Method	Release Function	Reference
Immunomagnetic bead (CellSearch System)	>75%	Immunofluorescence imaging	Yes	R1
Dielectrophoresis (DEP)	70–85%	Immunofluorescence imaging	Yes	R2
NanoVelcro CTC chip (1st-gen)	>95%	Immunofluorescence imaging	No	R3
NanoVelcro CTC chip (2nd-gen)	~87%	Immunofluorescence imaging	No	R4
NanoVelcro CTC chip (3rd-gen)	>90%	Immunofluorescence imaging	Yes	R5
CR-based NFAs	>90%	Immunofluorescence imaging	Yes	R6
**NF100**-based OECT	>90%	Immunofluorescence imaging/electrical signaling	Yes	This Work

Table S2. Comparison of EV production performance across various ES technologies.

EV Production Stimulation Platform/Method	EV Secretion (fold over control)	Detection Method	Cell Lines Used	Reference
Low-level Electrical Current	~1.7	NTA/BCA	B16F1/ 3T3	R7
Biphasic Electrical Pulse	0.38	NTA/BCA	CSC/ C-MSC	R8
Contractile Workload Electrical Stimulation	1.2	NTA/BCA	hiPSC	R9
Low-frequency Electrical Acupuncture	2	NTA/BCA	293HEK	R10
Cellular Nanoporation	~50	NTA/BCA/Imaging	MEFs/ BMDC	R11
**NF100**-based OECT	12.4	NTA/BCA	MCF7/ IBMSC	This Work

References:

[R1] Punnoose, E. A.; Atwal, S. K.; Spoerke, J. M.; Savage, H.; Pandita, A.; Yeh, R. F.; Pirzkall, A.; Fine, B. M.; Amler, L. C.; Chen, D. S.; Lackner, M. R., Molecular biomarker analyses using circulating tumor cells. *PLoS One* **2010**, 5(9) e12517.

[R2] Gascoyne, P. R.; Shim, S., Isolation of circulating tumor cells by dielectrophoresis. *Cancers* **2014**, 6(1) 545–579.

[R3] Wang, S.; Liu, K.; Liu, J.; Yu, Z. T. F.; Xu, X.; Zhao, L.; Lee, T.; Lee, E. K.; Reiss, J.; Lee, Y.-K.; Chung, L. W. K.; Huang, J.; Rettig, M.; Seligson, D.; Duraiswamy, K. N.; Shen, C. K.-F.; Tseng, H. R., Highly efficient capture of circulating tumor cells by using nanostructured silicon substrates with integrated chaotic micromixers. *Angew. Chem.* **2011**, 123(13) 3140–3144.

[R4] Hou, S.; Zhao, L.; Shen, Q.; Yu, J.; Ng, C.; Kong, X.; Wu, D.; Song, M.; Shi, X.; Xu, X.; OuYang, W.-H.; He, R.; Zhao, X.-Z.; Lee, T.; Brunicardi, F. C.; Garcia, M. A.; Ribas, A.; Lo, R. S.; Tseng, H. R., Polymer nanofiber-embedded microchips for detection, isolation, and molecular analysis of single circulating melanoma cells. *Angew. Chem.* **2013**, 125(12) 3463–3467.

[R5] Hou, S.; Zhao, H.; Zhao, L.; Shen, Q.; Wei, K. S.; Suh, D. Y.; Nakao, A.; Garcia, M. A.; Song, M.; Lee, T.; Xiong, B.; Luo, S.-C.; Tseng, H.-R.; Yu, H. H., Capture and stimulated release of circulating tumor cells on polymer-grafted silicon nanostructures. *Adv. Mater.* **2013**, 25(11) 1547–1551.

[R6] Chen, P. J.; Liu, R. Z.; Hsiao, Y. S., Self-assembled coronene nanofiber arrays: toward integrated organic bioelectronics for efficient isolation, detection, and recovery of cancer cells. *RSC Adv.* **2017**, 7(58) 36765-36776.

[R7] Fukuta, T.; Nishikawa, A.; Kogure, K., Low level electricity increases the secretion of extracellular vesicles from cultured cells. *Biochem. Biophys. Rep.* **2020**, 21, 100713.

[R8] Zhang, H.; Shen, Y.; Kim, I. M.; Liu, Y.; Cai, J.; Berman, A. E.; Nilsson, K. R.; Weintraub, N. L.; Tang, Y., Electrical stimulation increases the secretion of cardioprotective extracellular vesicles from cardiac mesenchymal stem cells. *Cells* **2023**, 12(6) 875.

[R9] James, V.; Nizamudeen, Z. A.; Lea, D.; Dottorini, T.; Holmes, T. L.; Johnson, B. B.; Arkill, K. P.; Denning, C.; Smith, J. G. W., Transcriptomic analysis of cardiomyocyte extracellular vesicles in hypertrophic cardiomyopathy reveals differential snoRNA cargo. *Stem Cells Dev.* **2021**, 30(24) 1215–1227.

[R10] Su, Z.; Yuan, Y.; Yu, M.; Liu, Y.; Klein, J. D.; Wang, X. H., Electrically stimulated acupuncture increases renal blood flow through exosome-carried miR-181. *Am. J. Physiol.-Renal Physiol.* **2018**, 315(6) F1542–F1549.

[R11] Yang, Z., Shi, J., Xie, J., Wang, Y., Sun, J., Liu, T., Zhao, Y., Zhao, X., Wang, X., Ma, Y., Malkoc, V., Chiang, C., Deng, W., Chen, Y., Fu, Y., Kwak, K. J., Fan, Y., Kuang, C., Yin, C., Rhee, J., Bertani, P., Otero, J., Lu, W., Yun, K., Lee, A. S., Jiang, W., Teng, L., Kim, B. Y. S., Lee, L. J., Large-scale generation of functional mRNA-encapsulating exosomes via cellular nanoporation. *Nat. Biomed. Eng.* **2020**, 4(1) 69–83.
